# scMAGeCK links genotypes with multiple phenotypes in single-cell CRISPR screens

**DOI:** 10.1186/s13059-020-1928-4

**Published:** 2020-01-24

**Authors:** Lin Yang, Yuqing Zhu, Hua Yu, Xiaolong Cheng, Sitong Chen, Yulan Chu, He Huang, Jin Zhang, Wei Li

**Affiliations:** 1 Center for Genetic Medicine Research, Children’s National Hospital, 111 Michigan Ave NW, Washington, DC 20010 USA; 2 Department of Genomics and Precision Medicine, George Washington University, 111 Michigan Ave NW, Washington, DC 20010 USA; 3 Department of Biochemistry & Molecular Medicine, George Washington University, 2300 Eye St., NW, Washington, DC 20037 USA; 4 Center for Stem Cell and Regenerative Medicine, Department of Basic Medical Sciences, and The First Affiliated Hospital, Zhejiang University School of Medicine, Hangzhou, 310058 Zhejiang China; 5 Institute of Hematology, Zhejiang University, Hangzhou, 310058 Zhejiang China; 6 Zhejiang University-University of Edinburgh Institute, Zhejiang University School of Medicine, Haining, 314400 Zhejiang China

## Abstract

We present scMAGeCK, a computational framework to identify genomic elements associated with multiple expression-based phenotypes in CRISPR/Cas9 functional screening that uses single-cell RNA-seq as readout. scMAGeCK outperforms existing methods, identifies genes and enhancers with known and novel functions in cell proliferation, and enables an unbiased construction of genotype-phenotype network. Single-cell CRISPR screening on mouse embryonic stem cells identifies key genes associated with different pluripotency states. Applying scMAGeCK on multiple datasets, we identify key factors that improve the power of single-cell CRISPR screening. Collectively, scMAGeCK is a novel tool to study genotype-phenotype relationships at a single-cell level.

**Electronic supplementary material:**

The online version of this article (10.1186/s13059-020-1928-4) contains supplementary material, which is available to authorized users.

## Introduction

Pooled genetic screens based on CRISPR/Cas9 genome engineering system is a widely used method to study the functions of thousands of genes or non-coding elements in one single experiment [1–3]. Recent CRISPR screening combined with single-cell RNA-seq (scRNA-seq) provides a powerful method to monitor gene expression changes in response to perturbation at a single-cell level. These technologies, including Perturb-seq [4, 5], CRISP-seq [6], Mosaic-seq [7], and CROP-seq [8], enabled a large-scale investigation of gene regulatory networks, genetic interactions, and enhancer-gene regulations in one experiment.

CRISPR screening coupled with scRNA-seq, which will be referred to as “single-cell CRISPR screening”, enables detecting the expression changes of whole transcriptome at a single-cell level. One can potentially search for perturbed genomic elements that lead to the differential expression of certain gene of interest. This approach resembles a fluorescence-activated cell sorting (FACS) experiment, where single cells are separated into groups of high (or low) expression of certain marker. Such “virtual FACS” experiment [7] can be performed on unlimited numbers of phenotypes, represented by the expressions of genes (or gene signatures). Therefore, single-cell CRISPR screening greatly eliminates the limitation of traditional screening experiment, where only one phenotype can be tested. However, few efforts were made to evaluate this approach, and no computational methods are available for the “virtual FACS” analysis based on single-cell CRISPR screening data.

Here we present scMAGeCK, a computational framework to systematically identify genes (and non-coding elements) associated with multiple phenotypes in single-cell CRISPR screening data. scMAGeCK is based on our previous MAGeCK models for pooled CRISPR screens [9–11], but further extends to scRNA-seq as the readout of the screening experiment. scMAGeCK consists of two modules: scMAGeCK-Robust Rank Aggregation (RRA), a sensitive and precise algorithm to detect genes whose perturbation links to one single marker expression, and scMAGeCK-LR, a linear-regression-based approach that unravels the perturbation effects on thousands of gene expressions, especially from cells that undergo multiple perturbations.

We demonstrated the ability of scMAGeCK to perform functional analysis from single-cell CRISPR screens. We applied scMAGeCK on public datasets generated from CROP-seq [8], a widely used protocol for single-cell CRISPR screening [12–14]. When compared with t-SNE clustering analysis, we found that for all the datasets, only one to two genes are enriched in clusters, while scMAGeCK identified many targets whose expressions are downregulated upon knockout with statistical significance. In the evaluation and comparison experiment, scMAGeCK demonstrates better specificity and sensitivity than other existing methods in analyzing single-cell CRISPR screens. Applying this approach to phenotypes, we identified oncogenic and tumor-suppressor genes and enhancers, by simply testing their associations with MKI67 (Ki-67), a commonly used proliferation marker. We further tested our scMAGeCK approach on mouse embryonic stem cells (mESCs) and identified key genes associated with different pluripotency states. These outcomes indicated that scMAGeCK enabled the reconstruction of a complex genotype-phenotype network.

Finally, we studied key factors that determine the statistical power of single-cell CRISPR screens. The efficiency of gene knockouts (or knockdowns) varies between different targets and different single cells. Highly expressed target genes tend to have a stronger effect of downregulation compared with moderately or lowly expressed targets. Screens with high multiplicity of infection (MOI), where multiple sgRNAs enter into one cell, have improved sensitivity and specificity compared with screens performed in low MOI.

## Results

### scMAGeCK method overview

We previously developed MAGeCK and MAGeCK-VISPR, two algorithms to model gene knockouts from genome-wide CRISPR/Cas9 screens [9, 10]. MAGeCK models the read counts of single-guide RNAs (sgRNAs) using a negative binomial (NB) distribution and prioritizes genes with a revised robust rank aggregation algorithm (alpha-RRA, [15]). The alpha parameter introduced in MAGeCK is used to determine significant and non-significant gRNAs. In addition, “MAGeCK-VISPR” models complex experimental designs using a generalized linear model and an expectation-maximization (EM) approach to optimize all the parameters.

**Fig. 1:**
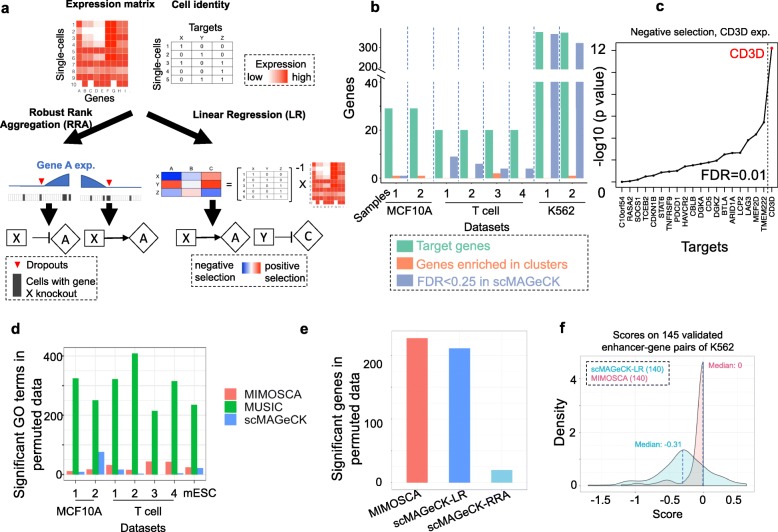
scMAGeCK pipeline and a comparison with clustering analysis and other methods on single-cell CRISPR screens. **a** An overview of the scMAGeCK pipeline. The input of scMAGeCK includes a scaled expression matrix of all genes in all single cells, together with cell identity information on the targets of each single cell. scMAGeCK includes two modules: RRA and LR. RRA infers gene regulatory relationship on certain gene expression (e.g., gene A) using the rankings of single cells and takes dropout events into consideration. LR infers the gene regulatory network on all possible gene expressions. **b** A comparison of scMAGeCK with clustering analysis on three different public CROP-seq datasets. The total number of target genes, genes that are enriched in certain cluster, and genes whose downregulation is considered as statistically significant (FDR < 0.25) are shown. Gene A is considered enriched in certain cluster are defined as single cells carrying gene A knockout consists of > 20% total cells in that cluster, and with adjusted *p* value smaller than 0.25 using chi-squared test. **c** The ranking of genes in reducing CD3D expression in the T cell CROP-seq dataset. **d** The significant GO terms (FDR < 0.05) in the permutated CROP-seq datasets as a measurement of false positives. **e** The significant genes (FDR < 0.05) of each method in the permutated CROP-seq datasets. For all the datasets, we randomly selected 50 expression markers and identified significant perturbations as a measurement of false positives. **f** The selection score distribution of scMAGeCK-LR and MIMOSCA over 145 validated enhancer-gene pairs in [13]. The number of pairs identified by each method is shown in parenthesis

scMAGeCK applies the statistical models of MAGeCK and MAGeCK-VISPR to single-cell CRISPR screening data. scMAGeCK includes two modules, scMAGeCK-RRA and scMAGeCK-LR (Fig. 1a). To identify genes whose perturbation associated with the expression of a gene of interest, scMAGeCK-RRA first ranks single cells according to the target gene expression. Next, scMAGeCK uses RRA to test whether single cells with particular gene perturbation are enriched in a higher (or lower) expression of the target. The alpha parameter is set to limit RRA on single cells whose marker expression is greater than zero, therefore minimizing the effect of possible dropout events. Another module, scMAGeCK-LR, simultaneously investigates the effects of all possible gene expressions. scMAGeCK-LR uses a linear regression model to calculate the “selection” score, similar to “log-fold change,” that describes the degree of perturbations (see “Methods” for more details).

scMAGeCK-RRA and scMAGeCK-LR provide two different approaches for single-cell CRISPR screening data. As scMAGeCK-RRA is a non-parametric test method, it is sensitive to detect subtle and non-linear expression changes. On the other hand, scMAGeCK-LR simultaneously tests the expressions of thousands of genes and is able to deal with cells targeted by multiple sgRNAs.

### Comparisons with clustering analysis and other algorithms

A typical approach to analyze perturbation effect in single-cell CRISPR screening is “enrichment by clustering”: users first cluster single cells based on their gene expression patterns, then check whether certain sgRNAs are enriched in one or more of these clusters using chi-squared or hypergeometric tests. We applied this approach to several public CROP-seq datasets performed on different cell types, including breast epithelial cells (MCF10A), unstimulated and stimulated primary human T cells, and myelogenous leukemia cells (K562) [12–14]. The number of perturbed genes or enhancers vary from around 20 (MCF10A and T cell) to over 1000 (K562). We found that the enrichment by clustering approach only identified one to two genes whose sgRNAs are enriched in certain clusters (Fig. 1b, Additional file 1: Figure S1). The small number of enriched targets in clusters, which also depends on the outcomes of clustering algorithms, limits downstream analysis, including the evaluation of knockout efficiency.

Instead of clustering analysis, we used scMAGeCK-RRA to investigate whether target gene knockout reduces their expressions. In two out of three datasets, we found that 25% (MCF10A data) and 95% (T cell data) of the target genes have reduced expressions with statistical significance, respectively, a demonstration that scMAGeCK-RRA better captures the effect of gene perturbation than the clustering analysis. For example, CD3D knockout strongly reduces CD3D expressions in single cells (Fig. 1c), while cells targeting CD3D are not enriched in any clusters (Additional file 1: Figure S1a-e).

We next compared scMAGeCK with two other methods, MIMOSCA [5] and MUSIC [16]. MIMOSCA uses a regularized linear model, similar with scMAGeCK-LR, to decompose gene expression matrix (from Perturb-seq) into a regulatory matrix, where the effect of sgRNAs on individual genes is modeled within. MUSIC uses the Topic Model, a method in natural language processing, to connect biological function (“topic”) to gene expression (“word”) in a single cell (“document”) under perturbation. A comparison of the features available for each method is presented in Table 1.

**Table 1:** A comparison of scMAGeCK with two available methods, MUSIC and MIMOSCA on different features

	scMAGeCK-RRA	scMAGeCK-LR	MUSIC	MIMOSCA
Analysis method	Rank based	Linear model	Topic model	Linear model
Permutation	*Y*	*Y*	N	*Y*
Test for certain expression-based phenotype	*Y*	*Y*	N	*Y*
Suitable for high MOI	N	*Y*	N	*Y*
Non-linear regulatory relationships	*Y*	N	NA	N
Use sgRNA-target information	*Y*	*Y*	*Y*	N
Use negative control	*Y*	*Y*	*Y*	N
R/Seurat support	*Y*	*Y*	N	N

The performances of these algorithms are evaluated based on three public CROP-seq datasets (MCF10A, T cell, and K562), as well as a new CROP-seq dataset we generated on mouse embryonic stem cells (mESCs). Since MUSIC is an unsupervised method to identify the biological functions of perturbed genes, we first systematically compared each method in terms of identifying enriched Gene Ontology (GO) terms associated with each perturbation. For each perturbed gene, we first permuted single-cell sgRNA labels and identified top genes with strongest expression changes and their enriched GO terms (see “Methods” for more details). Since the sgRNA labels of single cells are randomly shuffled, any significant term is considered as false positive. Among those, scMAGeCK-LR and MIMOSCA identified fewer enriched GO terms than MUSIC (Fig. 1d). scMAGeCK-LR has the fewest terms in six out of seven CROP-seq samples, demonstrating its good control of false positives.

To evaluate the sensitivity of three methods, we compared the enriched GO terms on the original CROP-seq datasets. Only terms that are found in at least two out of three methods are considered as “ground truth” terms (Additional file 1: Figure S2-S3), and their associated *p* values are compared across different methods. Three out of seven datasets have at least one strong GO term (*q* < 1e−4) identified by multiple methods (Additional file 1: Figure S2). Among these datasets, scMAGeCK achieved stronger enrichment, evidenced by lower *q* values (Additional file 1: Figure S2). For the rest of the datasets (Additional file 1: Figure S3), results vary by different methods. MUSIC has the strongest *q* values in some datasets (e.g., mESC and some T cell), possibly due to the fact that the comparisons are limited on MUSIC outputs (see “Methods”) and that MUSIC has a relatively high false positive rate (Fig. 1d).

These comparisons did not include scMAGeCK-RRA as scMAGeCK-RRA requires a specific expression marker as an input. To compare the false positive rate of using certain expression markers, we randomly selected expression markers (from protein-coding genes) in permutated CROP-seq datasets and identified statistically significant genes (FDR < 0.05) as a measurement of false positive (see “Methods” for more details). Three different methods that allow specific expression marker as input are compared: scMAGeCK-RRA, scMAGeCK-LR, and MIMOSCA (Fig. 1e). Both scMAGeCK modules demonstrated fewer levels of false positives than MIMOSCA, while scMAGeCK-RRA has the fewest number of significant genes as false positives.

The original K562 CROP-seq study used an independent approach to identify 145 canonical enhancer-gene pairs, where enhancer perturbations significantly altered target gene expressions [13]. We compared the corresponding enhancer-gene scores and *p* values between scMAGeCK and MIMOSCA (Fig. 1f and Additional file 1: Figure S4). The majority of these enhancer-gene pairs received negative scores from both methods, in agreement with the enhancer functions on these genes. Compared with scMAGeCK that outputs almost all of the canonical pairs, MIMOSCA fails to report many of the enhancer-gene pairs (Additional file 1: Figure S4b) or generates zero scores (Fig. 1f). Collectively, these comparisons demonstrated the good control of false positives and better sensitivity of scMAGeCK over other methods.

### Identification of known oncogenic and tumor-suppressor genes and enhancers

**Fig. 2:**
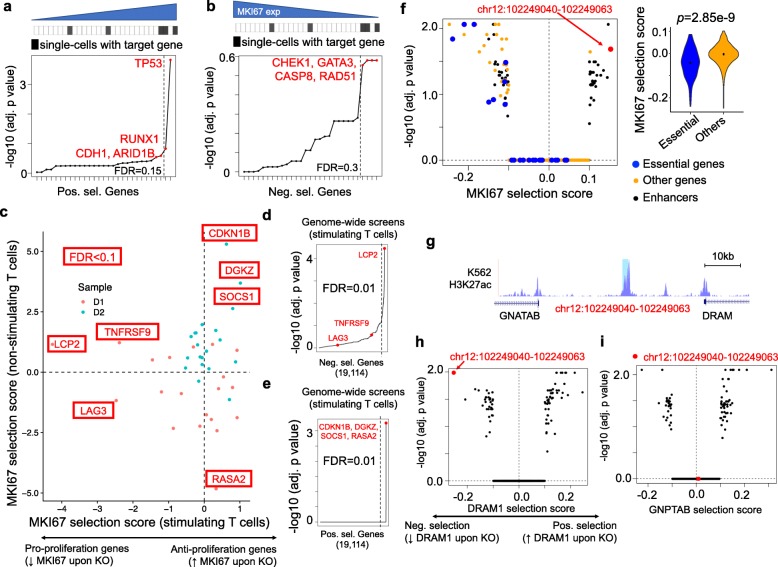
Associations with the expression of Ki-67 (MKI67), a proliferation marker. **a**, **b** The rankings of genes that are positively (**a**) or negatively selected (**b**) on MKI67 expression. Here, positive selection in **a** indicates single cells with certain target gene knockout (black rectangle on the top) have higher MKI67 expression. **c** The MKI67 selection score in stimulating and non-stimulating T cells in the T cell CROP-seq dataset. Genes performed in two different patient samples (D1/D2) are marked with different colors, and genes with FDR < 0.1 are highlighted. The selection score is calculated based on the *p* values reported from RRA, with direction depending on whether it is a positively (or negatively) selected genes. See “Methods” for more details. **d**, **e** The ranking of selected genes in **c** in genome-wide CRISPR screening, including negative selection ranking (**d**) and positive selection ranking (**e**). **f** The MKI67 selection score and *p* values calculated from scMAGeCK-LR in K562 dataset. Essential genes (ribosomal subunits and proteasomes) are marked in blue, while the tumor-suppressor-like enhancer of interest (chr12:102249040-102249063) is highlighted in red. Inset: the distribution of MKI67 selection score between essential genes and other genes. *p* value is calculated using Wilcox rank sum test. **g** The chromosome view of the enhancer chr12:102249040-102249063. **h**, **i** The DRAM1 and GNPTAB selection score and their corresponding *p* values. The enhancer chr12:102249040-102249063 is highlighted in red

We first used scMAGeCK-RRA to identify genes that modulate the expression of Ki-67 (*MKI67*), a commonly used marker for cell proliferation. In MCF10A CROP-seq, the knockout of *TP53* tumor-suppressor gene strongly induced MKI67 expression in corresponding single cells (adjusted *p* value = 1.5e−4; Additional file 1: Figure S5a). Other gene knockouts (*RUNX1*, *CDH1*, and *ARID1B*) have similar effect, consistent with their reported tumor-suppressor roles in breast cancer or other cancer types [17–19] (Fig. 2a). On the other hand, four gene knockouts significantly reduce Ki-67 expression (Fig. 2b). Among those, CHEK1 is a checkpoint kinase that is essential for normal and cancer cells (Additional file 1: Figure S5b) [20], GATA3 is a critical transcription factor with known oncogenic role [21], and RAD51 has been reported as an oncogene with elevated expression in multiple cancer types including breast cancer [22]. CASP8 has multiple functions in different contexts [23], with a possible essential role in breast cancer cell lines [24]. Many of these genes are consistent with their roles as tumor suppressors or oncogenes in genome-wide CRISPR and RNAi screens (Additional file 1: Figure S5c-d). Some genes have opposite roles compared with genome-wide CRISPR screens, an indication that they may function in a cancer type-specific manner.

In the T cell CROP-seq dataset, we identified different genes that regulate *MKI67* expression in non-stimulating and stimulating T cells (Fig. 2c) and compared their roles in genome-wide CRISPR screens in stimulating T cells, previously published in [14] (Fig. 2d, e). Here, we defined a “selection score” based on the *p* values calculated by scMAGeCK-RRA to describe the direction (and the degree) of *MKI67* regulation (see “Methods” for more details). Among those, four genes play anti-proliferation roles in stimulating T cells (*CDKN1B, DGKZ, SOCS1,* and *RASA2*). All these genes are top positively selected hits in genome-wide CRISPR screens (Fig. 2e). LCP2, the strongest negative selection hit in genome-wide screens (Fig. 2d), is also identified as the top pro-proliferation gene, consistent with its essential role in T cell function [14]. TNFRSF9 (CD137) is a co-stimulatory factor in T cells whose knockout reduces *MKI67* expression but is not identified in genome-wide CRISPR screens (Fig. 2d). In contrast, LAG3, an immune checkpoint receptor, paradoxically reduces MKI67 expression upon knockout, a demonstration that different platforms may provide different results.

We next studied the expression of Ki-67 in the K562 CROP-seq dataset, where each cell is targeted by an average of 20 sgRNAs [13]. scMAGeCK-LR is used for the analysis, as scMAGeCK-RRA is not suitable for cells targeted by multiple gRNAs. Overall, knocking down essential genes, including ribosomal subunits and proteasomes, reduced MKI67 expression (Fig. 2f), consistent with their critical roles in cell functions. Several enhancers are among the top candidates whose perturbation changed Ki-67 expression (Fig. 2f). Among those, chr12:102249040-102249063 a putative enhancer that negatively regulates Ki-67 expression. This enhancer is located in the intergenic region of chromosome 12 with strong H3K27ac signals, proximal to the transcription start site (TSS) of two protein-coding genes (*GNPTAB* and *DRAM1*, Fig. 2g). To further identify the target genes, we ranked all genes/enhancers based on their perturbation effects on *GNPTAB* and *DRAM1* expressions (Fig. 2h, i). chr12:102249040-102249063 is among the top hits on reducing the expression of *DRAM1* (but not *GNPTAB*). Indeed, *DRAM1* (DNA damage regulated autophagy modulator 1) is a tumor-suppressor gene with decreased expression in various tumors and is required for the induction of autophagy by the p53 pathway [25]. Collectively, these results demonstrated that oncogenic and tumor-suppressor genes (and enhancers) can be readily identified by testing their associations with Ki-67 using scMAGeCK.

MKI67 is a widely used marker for proliferation. To investigate the effect of different proliferation markers (or marker combinations), we systematically compared MKI67 with cyclin a (CCNA1/2) and cyclin E (CCNE1/2), two cyclin family members that regulate the cell cycle. In addition, we included one cell cycle-related gene signature from GSEA MSigDB database (pathway name: WHITFIELD_CELL_CYCLE_LITERATURE) [26]. We tested whether these markers are indicative of known genes (or validated genes) that regulate proliferation (e.g., TP53, CDKN1B, LCP2; Additional file 1: Figure S6a). For K562 dataset, 53 essential genes were identified from K562 CRISPR screening [27] whose TSS are targeted in the CROP-seq library. These gene are evaluated whether their knockdowns reduced proliferation marker expressions (Additional file 1: Figure S6b), and their enrichment among all genes/enhancers in the library using GSEA (Additional file 1: Figure S6c). Overall, gene signatures and MKI67 worked better than cyclins to identify known or validated proliferation-associated genes (with the only exception in K562 high MOI where more essential genes are identified from CCNA2/CCNE1). In contrast, the behaviors of cyclin genes vary across datasets: some cyclin genes work equally well or better than MKI67/signature (e.g., CCNE1 in high MOI K562), but none is served as a stable indicator of proliferation. Interestingly, some markers provide opposite directions in certain perturbations (e.g., DGKZ knockout; Additional file 1: Figure S7). These results indicate that various qualities of the datasets, compositions of cells at different stages, and the use of different markers may contribute to different aspects upon one single phenotype of cell proliferation.

### Investigating multiple phenotypes using scMAGeCK

**Fig. 3:**
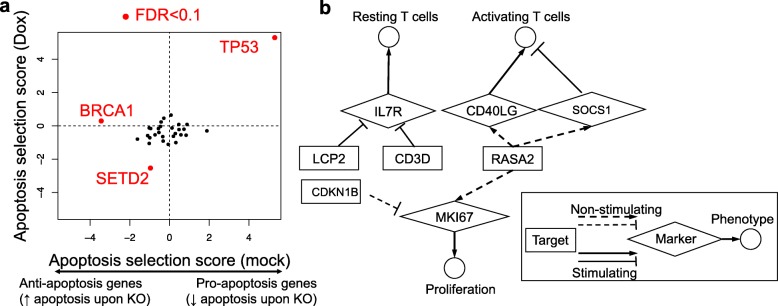
scMAGeCK on other phenotypes. **a** The apoptosis selection score of different genes in MCF10A CROP-seq datasets treated with doxorubicin (*y*-axis) and with mock control (*x*-axis). Genes with FDR < 0.1 are highlighted in red. Here the average expression of signature genes in an apoptosis gene set in MSigDB is served as a marker. The signature comes from genes that are downregulated in a breast cancer cell line (ME-A) undergoing apoptosis in response to doxorubicin (ID: GRAESSMANN_APOPTOSIS_BY_DOXORUBICIN_DN). **b** The target-marker-phenotype network in T cell CROP-seq dataset. Target genes are genes that are screened in CROP-seq dataset, while markers that are known to be associated with resting T cells, activating T cells, and proliferation are selected. Gene regulatory relationship from non-stimulating and stimulated cells is shown in dashed and solid lines, respectively. Genes are selected based on the FDR cutoff 0.1 from scMAGeCK-RRA

We set out to use scMAGeCK to study multiple phenotypes beyond proliferation. In MCF10A CROP-seq dataset, we studied the effect of gene knockouts on apoptosis, as doxorubicin is known to induce apoptosis in normal and tumor cells [28]. We used the average expression of genes in an apoptosis signature in the MSigDB database [26] as the readout. These genes are downregulated in a breast cancer cell line (ME-A) undergoing apoptosis in response to doxorubicin [29], a system mostly resemble the experimental conditions in MCF10A CROP-seq. Under the false discovery rate 0.1 cutoff, we found three genes that significantly modulate the expressions of apoptosis signatures in two conditions (doxorubicin treatment or mock treatment, Fig. 3a). Among those, *TP53* consistently served as a pro-apoptosis gene, consistent with its critical role in apoptosis. Interestingly, BRCA1 serves as an anti-apoptosis gene in the normal MCF10A cells, consistent with previous reports that BRCA1 loss triggers apoptosis and BRCA1 deletion causes growth inhibition in MCF10A [30, 31].

In T cell CROP-seq data, we chose known markers that are associated with multiple phenotypes in T cells, including resting (IL7R), activating T cells (CD40LG, SOCS1), and proliferation (MKI67). The outputs of scMAGeCK enabled an unbiased construction of genotype-phenotype network in non-stimulating and stimulating T cells (Fig. 3b). Among these, LCP2 and CD3D knockout significantly increases IL7R, consistent with their essential roles in T cell stimulation. Some genes may have opposite roles in different conditions; for example, RASA2 is a positive regulator of MKI67 in non-stimulating cells (Fig. 3b). On the other hand, genome-wide screens on stimulating T cells revealed RASA2 as a negative regulator (Fig. 2e). This is consistent with CROP-seq data, although the FDR is not significant (0.37 in stimulating T cells; Fig. 2c). This genotype-phenotype network provides an intuitive approach to study gene functions in different contexts.

### scMAGeCK identified key genes associated with different pluripotency states of embryonic stem cells

**Fig. 4:**
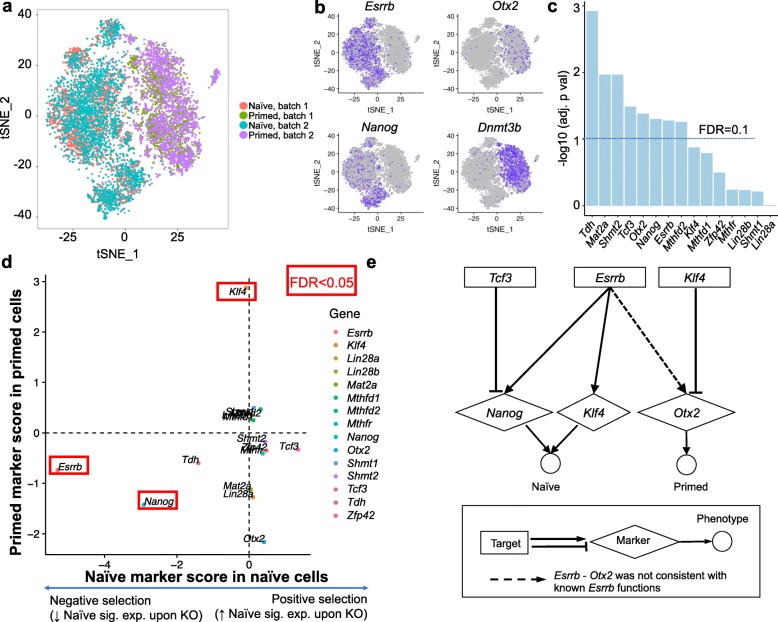
CROP-seq on mouse embryonic stem cells (mESC) uncovered known regulators for stem cell differentiation. **a** The t-SNE plot on single-cell expression profiles in naïve or primed states in two batches. **b** Selected marker expression, including *Esrrb*, *Nanog* (naïve markers), *Otx2*, and *Dnmt3b* (primed markers). **c** The adjusted *p* values, calculated from scMAGeCK-RRA, on target gene downregulation. **d** The naïve marker scores as well as primed marker scores in their corresponding cell states. Genes with FDR < 0.05 are highlighted in red. Naïve marker score is based on the average expression of four naïve marker genes: *Nanog*, *Esrrb*, *Klf4*, and *Tdh*, while primed marker score is based on *Otx2* expression. **e** The target-marker-phenotype network constructed from scMAGeCK results. Only significant results (FDR < 0.01) are used to construct the network. Dotted arrow indicates *Esrrb-Otx2* that is inconsistent with known *Esrrb* functions

Having demonstrated the ability of scMAGeCK to perform functional analysis of multiple phenotypes, we performed CROP-seq experiments to interrogate genes that are critical for mouse embryonic stem cell (mESC) pluripotency and differentiation. The pluripotent state of the mESCs is highly dynamic, including a more primitive naïve state and a primed state ready for differentiation [32]. As they represent two key different developmental stages of pre- and post-implantation embryos, it is important to understand what factors regulate these two states. We thus designed 45 guides to perturb 15 genes including naive and primed pluripotency-associated transcription factors and metabolic genes. CROP-seq experiments were performed with samples in the two states of mESC (naïve and primed), respectively (see “Methods”). Overall, we obtained the transcriptome profiles of ~ 2000 cells per sample using the InDrop platform [33]. t-distributed stochastic neighbor embedding (t-SNE) clustering demonstrated a clear separation of both states, not batches (Fig. 4a). Known markers are selectively expressed in each state, including *Nanog* in the naïve state, and *Dnmt3b* in the primed state (Fig. 4b), respectively.

Consistent with the results from public CROP-seq datasets, clustering analysis only identified two sgRNAs from two genes that are enriched in certain clusters (Additional file 1: Figure S8). In contrast, scMAGeCK-RRA identified 8 out of 15 genes whose expression is reduced upon knockout with statistical significance (Fig. 4c). For the remaining seven genes that do not reach FDR threshold (0.1), six have less than 100 supporting single cells. The small number of single cells, together with other reasons (e.g., low sgRNA efficiency, gene knockout does not change their expression), may contribute to the “failure” to detect target gene downregulation. Interestingly, knocking out Lin28a/b did not change their expressions, but two gRNAs led to some enrichment effect on a subset of cells (Additional file 1: Figure S8), possibly due to the potential off-target effects of these sgRNAs.

We next investigated the effect of individual gene knockout on both states, using the expression of known naïve and primed markers. To this end, we used the expression of *Otx2*, a primed state-specific gene [34] and a combined expression of *Nanog*, *Esrrb*, *Klf4*, and *Tdh*, four naïve markers, as the readout [35]. The scores of both markers are shown for naïve and primed cells, respectively (Fig. 4d). Among those, *Nanog* knockout significantly reduced the naïve marker expression, consistent with its critical role in naïve pluripotency [36]. *Esrrb* knockout decreases, whereas *Tcf3* knockout increases, the naive marker expressions, consistent with the previous report that *Tcf3* inhibits naïve state through *Esrrb* [37]. In the primed state sample, *Klf4* knockout increases primed markers, demonstrating its role in maintaining naive state and preventing differentiation [38].

Based on the known functions of perturbed genes, we built a target-marker-phenotype network that describes the gene regulatory network in both cell types (Fig. 4e). The inputs of the network analysis are scMAGeCK-RRA results, using a set of predetermined expression markers that are known to each state (i.e., *Nanog* and *Klf4* for naïve state, and *Otx2* for primed state in Fig. 4e). Target-marker associations with statistical significance (FDR < 0.01) are used to draw the network. This network, constructed unbiasedly from CROP-seq data, includes previously reported naive and primed regulatory relations. For example, *Tcf3* regulates mES pluripotency through suppressing *Naong* expression [39]. *Klf4* may restrain Otx2 expression, which is supported from evidences that *Otx2* downregulates *Klf4* and *Klf* absence inhibits *Nanog* [40]. On the other hand, the *Esrrb*-*Otx2* regulation in primed state is not consistent with the known function of *Esrrb*, as *Esrrb* plays a critical role in maintaining naive pluripotency as a direct target of *Nanog* [41] and *Otx2* suppress the expression *Nanog* [42]. In summary, the scMAGeCK generated network provides opportunities to unbiasedly identify known and novel regulations.

### High target expression and high MOI improves the power of single-cell CRISPR screening

**Fig. 5:**
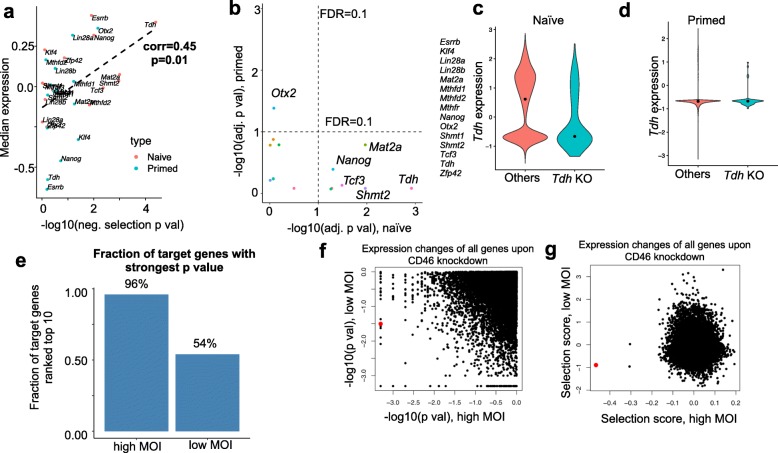
Factors that determine knockout efficiency in single-cell CRISPR screens. **a** The knockout effect on target gene downregulation (measured by negative selection *p* value) and the median target expression in mESC dataset. **b** The negative selection *p* values of all targets in naïve and primed states in mESC CROP-seq dataset. **c**, **d** The expression of *Tdh* in *Tdh* knockout cells and other cells in naïve and primed states. The CD46 gene is marked as red. **e** The fraction of target genes with the strongest *p* values, where target gene is ranked top ten among all other genes, in high MOI and low MOI conditions. **f**, **g** The *p* value (**f**) and selection score (**g**) of all possible genes upon CD46 knockdown in K562 dataset. CD46 itself is marked as red

We set out to determine factors that affect the statistical power of single-cell CRISPR screening. We first determine whether the expression of target gene is reduced in corresponding single cells, an indication of target knockout efficiency. Different levels of downregulation are observed in different datasets and samples (Fig. 1b). Overall, we observed a strong correlation between the effect of downregulation (measured by the negative selection *p* values from scMAGeCK) and median gene expression in all datasets (Fig. 5a, b, Additional file 1: Figure S9). Genes that are highly expressed are more likely to have a strong downregulation. For example, in mESC CROP-seq dataset, targets may undergo different downregulation effects in different states (Fig. 5b). *Tdh*, a highly expressed gene in naïve but not in primed cells, demonstrates strong downregulation effect only in the naïve state (Fig. 5c, d).

Some CRISPR screening and single-cell CRISPR screening studies suggested using high multiplicity of infection (MOI) to increase the power of screening [13, 43]. We set out to compare the effect of high vs. low MOI in terms of a target gene knockout effect using scMAGeCK. In the K562 dataset, the screening is performed in two different conditions, one with high MOI (with around 28 gRNAs per cell) and the other with low MOI (around 1 gRNA per cell). We evaluated the statistical power of both conditions, by looking at the effect of downregulation in over 300 protein-coding genes. The selections scores of these genes are highly correlated between two conditions (Additional file 1: Figure S10a). However, over 95% of the target genes are among the strongest downregulated genes in high MOI screen, while only 50–60% of them ranked top in low MOI screen (Fig. 5e, Additional file 1: Figure S10b). For example, CD46 has the strongest downregulation for CD46 perturbation in high MOI, but only ranks 876th (out of 12,000 genes) in terms of *p* value, and 305th in terms of selection score in low MOI condition, respectively (Fig. 5f, g). This comparison demonstrates that a better statistical power can be obtained by increasing the level of MOI in the screening experiment.

Across different CROP-seq datasets, K562 performed better than the others (Fig. 1b) as both conditions reached higher numbers of downregulated genes upon knockdown. Two possible reasons may contribute to the high successful rate. First, CRISPR inhibition (CRISPRi) was used in K562 datasets to directly knock down the expression of target gene expression. In contrast, others datasets use CRISPR-Cas9 to knock out target gene, whose expression may not be affected [12]. Second, genes selected in the K562 screening generally have high expression in K562 cell line [13], a factor that contributes to the high success rate (Fig. 5). Third, high MOI increases the number of single cells per target gene, one reason that the statistical power is improved compared with low MOI. Collectively, these results indicate that to reach a better knockdown effect in CROP-seq experiments, users may select genes with moderate or high expressions and increase the number of cells per each gene perturbation (by increasing MOI).

## Discussion

CRISPR screening using single-cell RNA-seq as readout (“single-cell CRISPR screening”) is a promising technology that overcomes several limitations of traditional CRISPR screening. First, it enables an interrogation of genotypes on potentially unlimited numbers of phenotypes, represented by the expressions of genes or gene signatures. In contrast, CRISPR screening only studies one single phenotype of cell viability or reporter expression. Second, single-cell CRISPR screening reports the effect of perturbations at the single-cell level, compared with traditional CRISPR screening that are often performed on bulk cells. To this end, scMAGeCK expands our previous MAGeCK algorithmic framework to analyze single-cell CRISPR screening data, providing a powerful computational tool to link genotypes with multiple phenotypes. The two modules of scMAGeCK provide complementary tools to study gene perturbation in different contexts. scMAGeCK-RRA is an algorithm that reaches the lowest false positive rate (Fig. 1e) and is able to detect subtle, non-linear expression changes that scMAGeCK-LR is not able to identify. For example, scMAGeCK-LR failed to detect CHEK1 (score = − 0.06, adjusted *p* value = 0.93) whose knockout reduces MKI67 expression in only a small fraction of cells (Additional file 1: Figure S5b), which is readily identified as the top hit in scMAGeCK-RRA (Fig. 2b). In contrast, scMAGeCK-LR provides a convenient tool to model the expressions of all genes and deals with cells infected by multiple sgRNAs, where scMAGeCK-RRA may fail (e.g., in Additional file 1: Figure S11).

We tested scMAGeCK on several public CROP-seq experiments. scMAGeCK identified potential oncogenes and tumor-suppressor genes (and enhancers) by simply testing their associations with the expression of Ki-67, a proliferation marker. We demonstrated the ability of scMAGeCK to study other phenotypes, including apoptosis, T cell stimulation, stem cell differentiation, etc. These results generated from scMAGeCK enabled an unbiased reconstruction of genotype-phenotype network, providing an intuitive picture for users to study gene regulatory network and enhancer-gene regulations.

So far, CRISPR screen studies on mESC pluripotency or naive and primed state transition is mainly based on genetically labeled fluorescence reporters as readout [44–46], which is limited by only one or two genes. Here we employed a single-cell RNA-seq combined with CRISPR screening technology (CROP-seq) and used whole-cell transcriptome as readout of cell fate changes. With the aid of scMAGeCK, we were able to capture alteration of cell fate defined by a combination of marker genes upon genetic perturbation and to build or refine the regulatory network of mESCs.

Some single-cell CRISPR screening technologies (Perturb-seq, CRISP-seq, MOSAIC-seq) use additional barcodes to determine the single-cell identity. The sgRNA-barcode correspondence may be compromised during the screening process, which may complicate downstream analysis results [47–49]. Here, we exclusively focus on CROP-seq where sgRNA itself serves as the barcode. Once the sgRNA-barcode issue is solved with improved protocol, scMAGeCK will be extended to other platforms as well.

Most of the CROP-seq datasets based on target gene knockout have a low successful rate (Fig. 1b). There may be various reasons to the failures, including (1) low target gene expression, (2) low guide knockout efficiency, and (3) not enough single cells to reach statistical significance. Therefore, to increase the success rate, users may pick up genes with moderate or high expression and/or ensure that there are enough number of cells for the analysis (e.g., by increasing MOI). One caveat of this approach is that target gene knockout may not necessarily reduce its expression (Hill et al. 2018). To overcome this limitation, one may look at the expression of known downstream targets or switch to CRISPR inhibition instead of CRISPR knockout to directly repress target gene expression. As more CROP-seq (or other types of single-cell technology) datasets accumulate, we may be able to study how guide knockout efficiency affects the outcome of the screen.

Compared with CROP-seq using low MOI condition, high MOI reaches a better performance in terms of target gene knock down (Fig. 5d–f). However, high MOI condition may not be suitable for CRISPR knockout based CROP-seq, as multiple DNA cleavage within single cell may induce strong DNA damage response in the cells [27, 50, 51]. Further investigations are needed to determine the best MOI for CROP-seq based on CRISPR/Cas9 gene knockouts.

As the quality of different CROP-seq datasets varies (e.g., Fig. 1b), choosing a proper false discovery rate (FDR) cutoff is an essential step. The choice of appropriate FDR depends on how stringent the users would like the results would be. Users may select low thresholds (e.g., 0.01) if they want fewer but more reliable results, and high thresholds (e.g., 0.25) if more results are needed and a high false positive rate can be tolerated.

Besides scRNA-seq, single-cell epigenomic profiling could serve as the screening readout (e.g., single-cell ATAC-seq), providing a novel approach to measure epigenome changes upon perturbation [52]. In the future, scMAGeCK will support other types of single-cell sequencing data as the screening readout, enabling analysis on phenotypes beyond gene expression.

## Methods

### The scMAGeCK algorithm

scMAGeCK consists of two modules, scMAGeCK-RRA and scMAGeCK-LR, based on our previous MAGeCK and MAGeCK-VISPR algorithms [15]. scMAGeCK-RRA first ranks single cells based on the expression of gene A of interest. Then, the RRA algorithm proposed by Kolde et al. [12] to evaluate whether single cells bearing certain gene X is enriched in the front of the ranked list*.* Suppose *M* single cells are ranked in the experiment according to gene A expression in the descending order, *R* = (*r*_1_, *r*_2_, …, *r*_*n*_) is the vector of ranks of *n* single cells targeting gene X (*n* < < *M*, *r*_*i*_ ≤ *M* where *i* = 1, 2, …, *n*), and *α* is the percentage of single-cells that have non-zero counts on gene A. We first normalize the ranks into percentiles *U* = (*u*_1_, *u*_2_, …, *u*_*n*_), where *u*_*i*_ = *r*_*i*_/*M*(*i* = 1, 2, …, *n*). Under null hypotheses where the percentiles follow a uniform distribution between 0 and 1, the *k*th smallest value among *u*_1_, *u*_2_, …, *u*_*n*_ is an order-statistic which follows a beta distribution *B*(*k*, *n*, + 1 − *k*). RRA computes a *p* value *ρ*_*k*_ for the *k*th smallest value based on the beta distribution.

For positive selection (cells with gene X knockout are enriched in higher A expression), the significance score of the gene, the *ρ* value, is defined as *ρ* = min(*p*_1_, *p*_2_, …, *p*_*j*_), where *j* out of the *n* single cells targeting gene X have non-zero read count on gene A. For negative selection, single cells that are ranked in the front will have zero counts (dropouts). Therefore, we calculated *ρ* = min (*p*_j + 1_, *p*_j + 2_, …, *p*_*n*_) where the first *j* single cells have zero counts on gene A (and are excluded from the calculation of *ρ*).

To compute a *p* value based on the *ρ* values, we performed a permutation test where the sgRNAs are randomly assigned to single cells. We then compute the FDR from the empirical permutation *p* values using the Benjamini-Hochberg procedure.

The selection score of gene X perturbation on gene A, calculated from scMAGeCK-RRA, combines the results of both negative and positive selection:

$$ {s}_{XA}=\left\{\begin{array}{c}\log {p}_{\mathrm{neg}}, if\ {p}_{\mathrm{neg}}<{p}_{\mathrm{pos}}\\ {}-\log {p}_{\mathrm{pos}}, if\ {p}_{\mathrm{pos}}<{p}_{\mathrm{neg}}\end{array}\right. $$

where *p*_neg_ and *p*_pos_ are the *p* values of negative selection and positive selection of perturbing gene X on gene A expression, respectively.

scMAGeCK-LR uses a linear regression model to calculate the selection scores of all genes. Let *Y* be the *M × N* expression matrix of *M* single cells and *N* genes. Let *D* be the *M × K* binary cell identity matrix, where *d*_*jX*_ = 1 if single cell *j* contains sgRNAs targeting gene *X* (*j* = 1, 2, …, *M*; *X* = 1, 2, …, *K*), and *d*_*jX*_ = 0 otherwise. The effect of target gene knockout on all expressed genes is indicated in a selection score matrix *S* with size *K × N*, where *s*_*XA*_ > 0 (<0) indicates gene *X* is positively (or negatively) selected on gene *A* expression, respectively. In other words, gene *X* knockout increases (or decreases) gene *A* expression if *s*_*XA*_ > 0 (<0), respectively.

The expression matrix *Y* is modeled as follows:

$$ Y=D\times S+\epsilon $$

where *ϵ* is a noise term following a Gaussian distribution with zero means. The value of *S* can be estimated using ridge regression:

$$ S={\left({D}^TD+\lambda I\right)}^{-1}{D}^TY $$

where *I* is the identity matrix, and *λ* is a small positive value (default 0.01).

To compute the empirical *p* value, we performed a permutation test similar with scMAGeCK-RRA, where the sgRNAs are randomly assigned to single cells. The FDR is then calculated using the Benjamini-Hochberg procedure.

### Public CROP-seq datasets

We used three public CROP-seq datasets. The MCF10A CROP-seq dataset [12], T cell CROP-seq dataset [14], and K562 CROP-seq dataset [13] are downloaded from Gene Expression Omnibus. All datasets are profiled through the 10X Genomics platform. Raw expression matrix from cellranger pipeline is imported and processed using Seurat pipeline (version 3.0) [53]. Briefly, single cells are first filtered out if they contain < 500 expressed genes or > 10% read counts coming from mitochondria genes. The expressions of the remaining cells are normalized and scaled based on the number of UMIs and mitochondrial gene expressions. The principal component analysis (PCA), clustering analysis, and t-SNE visualization are performed using default Seurat parameters.

### Comparisons with other methods

MUSIC is an unsupervised method that only outputs gene rankings and enriched Gene Ontology (GO) terms for each topic and is not able to rank genes based on certain expression-based phenotype. To accommodate the output of MUSIC, we generated enriched GO terms for scMAGeCK-LR and MIMOSCA as follows. We first selected the top gene *G* of each topic *T* generated by MUSIC. Then, we ranked all protein-coding genes based on their absolute selection scores of *G* in scMAGeCK-LR and MIMOSCA, chose *k* top genes (*k* is the number of genes in *T*), and use clusterProfiler to identify enriched GO terms. For consistency, we used clusterProfiler [54] to calculate the enriched GO terms for all three methods.

For permutated CROP-seq data, we randomly shuffled sgRNA-single cell relationship and run scMAGeCK-LR, MUSIC, and MIMOSCA afterwards. The permutation was repeated 10 times. Seven CROP-seq datasets are used (MCF10A, T cell and mESC). K562 was excluded since MUSIC was not able to run on both K562 datasets.

scMAGeCK-RRA, scMAGeCK-LR, and MIMOSCA are further compared using randomly selected genes as expression markers. For each permuted dataset, we randomly selected 50 protein-coding genes as markers and use three different approaches to identify statistically significant perturbations as a measurement of false positives (Fig. 1e).

### gRNA library construction

gRNA cassettes were ligated to CROP-seq-guide-puro vector using Gibson assembly with a ratio of 20:1 at 50 °C for 1 h, then dialyze the reaction against water. Electroporate the gRNA library to lucigen endura cells (Lucigen cat. no. 60242–2) using Lonza 2B nucleofector bacteria program 3. After transformation, add 1 ml pre-warmed Recovery Medium (Lucigen) and at 37 °C for 1 h while shaking at 225 rpm. Then 1 ml bacterial solution was plated on 25 cm × 25 cm ampicillin LB-agar dish at 34 °C for 18 h, then LB medium was added to collect the bacteria. Plasmid DNA was extracted with Tiangen EndoFree maxi Plasmid extraction kit (Tiangen cat. no. DP117).

### Lentivirus production for CROP-seq screens

HEK293T cells were plated onto 10-cm dishes at 6 million cells per dish in 10 ml of lentivirus packaging medium (Opti-MEM I (Gibco), 5% FBS (Gibco), 200 mM sodium pyruvate (Gibco)). Next day, HEK293T were transfected 11.7 μg constructed CROP-seq-guide-puro (containing gRNA library) with lipofectamine 3000 (Invitrogen) using two packaging plasmids psPAX2 (addgene 12260) and pMD2.G (addgene 12259). The medium was changed to lentivirus packaging medium 6 h after transfection. Viral containing supernatant were collected at 24 and 48 h. Viruses were filtered through a 0.22-μm filter and 10% PEG 6000 was added to concentrate CROP-seq virus. Then CROP-seq virus were placed at 4 °C overnight. Centrifuging 30 min at 4200 rpm, discarding the supernatant, and resuspending the CROP-seq virus with 500 μl PBS were done.

### Cell culture

Naive mouse ESCs were cultured in 2i/LIF medium (1:1 DMEM/F12 (Gibco) and neurobasal medium (Gibco) containing 1%(v/v) N2 and B27 supplements (Gibco), 1 mM PD03259010 (stem cell), 3 mM CHIR99021 (stem cell), 1000 U/ml mLIF (Peprotech), 1× l-glutamine (Gibco), 100 mM 2-mercaptoethanol (Sigma), and 1% penicillin-streptomycin (Gibco)) on 0.1% gelatin-coated dishes with MEF feeders. After transducing CROP-seq gRNA library, ESCs were transferred to FGF2/Activin DMEM/FBS medium (1:1 DMEM/F12 and Neurobasal medium containing 1%(v/v) N2 and B27 supplements, 10 ng/ml FGF-2 (Peprotech), 20 ng/ml Activin A (Peprotech), 1× l-glutamine, 100 mM 2-mercaptoethanol, and 1% penicillin-streptomycin) for 48 h to become primed state cells.

### Single-cell RNA-seq

Single-cell RNA sequencing was performed with 1cell-bio inDrop platform (Klein, Mazutis et al. 2015). In brief, cells were prepared in 1× PBS containing 1% volume/volume FBS with an input concentration of 40–60 cells/μl. A total of ~ 6000 cells were captured per sample with different microdevice flow rate conditions with BHM phase varying from 40 to 60 μl/h. Photo-cleavable barcoding oligos were released from barcoded hydrogel microspheres (BHMs) with exposed the collected droplets to UV (6.5 J/cm^2^ at 365 nm). Library preparation was carried out with in vitro transcription (IVT), followed by first PCR amplification with the following program before fragmentation: 1 cycle of 98 °C for 1 min, 10 cycles of 98 °C for 7 s, 60 °C for 30 s, 72 °C for 90 s, and 1 cycle of 72 °C for 3 min. Second PCR was conducted for final library amplification with following program: 1 cycle of 98 °C for 2 min, 2 cycles of 98 °C for 20 s, 55 °C for 30 s, 72 °C for 2 min, 9 cycles of 98 °C for 20 s, 65 °C for 30 s, 72 °C for 2 min, and 1 cycle of 72 °C for 5 min. One lane was used for sequencing both two samples on Hiseq X.

## Supplementary information

Additional file 1 Supplementary figures, including Figure S1-S11 (PDF 3755 kb)

Additional file 2 Review history (DOCX 936 kb)
