# Supplementary material for: scMAGeCK links genotypes with multiple phenotypes in single-cell CRISPR screens
Source: Genome Biol. 2020 Jan 24;21:19. doi: 10.1186/s13059-020-1928-4 (PMC6979386; doi:10.1186/s13059-020-1928-4)
Supplement: Supplementary file 1 — Supplementary figures, including Figure S1-S11 (PDF 3755 kb) [file 13059_2020_1928_MOESM1_ESM.pdf]

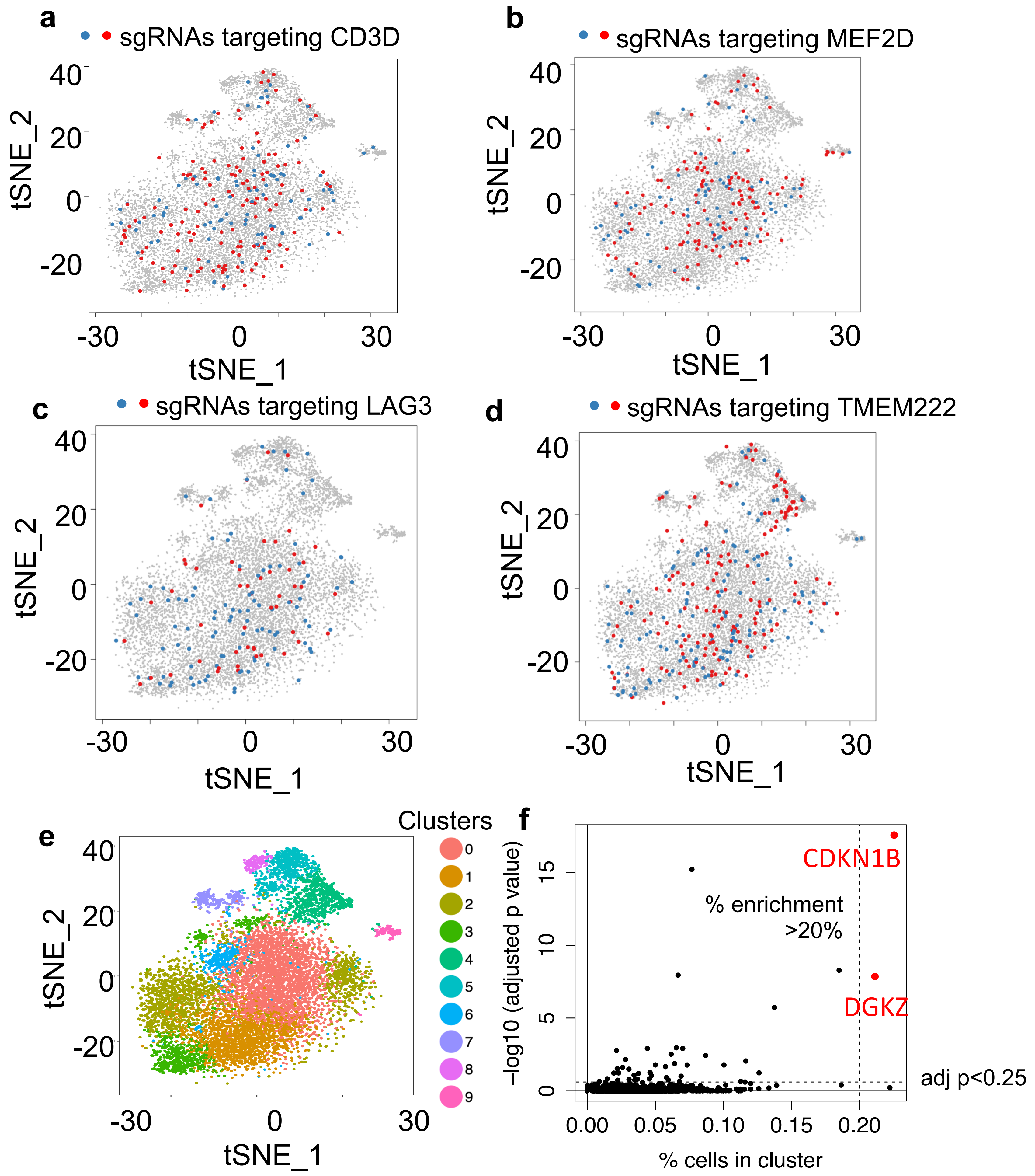

Figure S1: (a-d) t-SNE clustering of all single cells in T cell dataset. Dots with different colors represent different sgRNAs targeting that gene. (e), the clustering of all single cells in the T cell CROP-seq dataset using t-Distributed Stochastic Neighbor Embedding (t-SNE) analysis. (f) The “enrichment by clustering” approach to test the enrichment of cells of certain gene knockout in certain clusters. The percentage of cells in every cluster bearing certain gene knockout (x axis) is plotted against the adjusted p value calculated from chi-squared test (y axis) in T cell CROP-seq dataset.

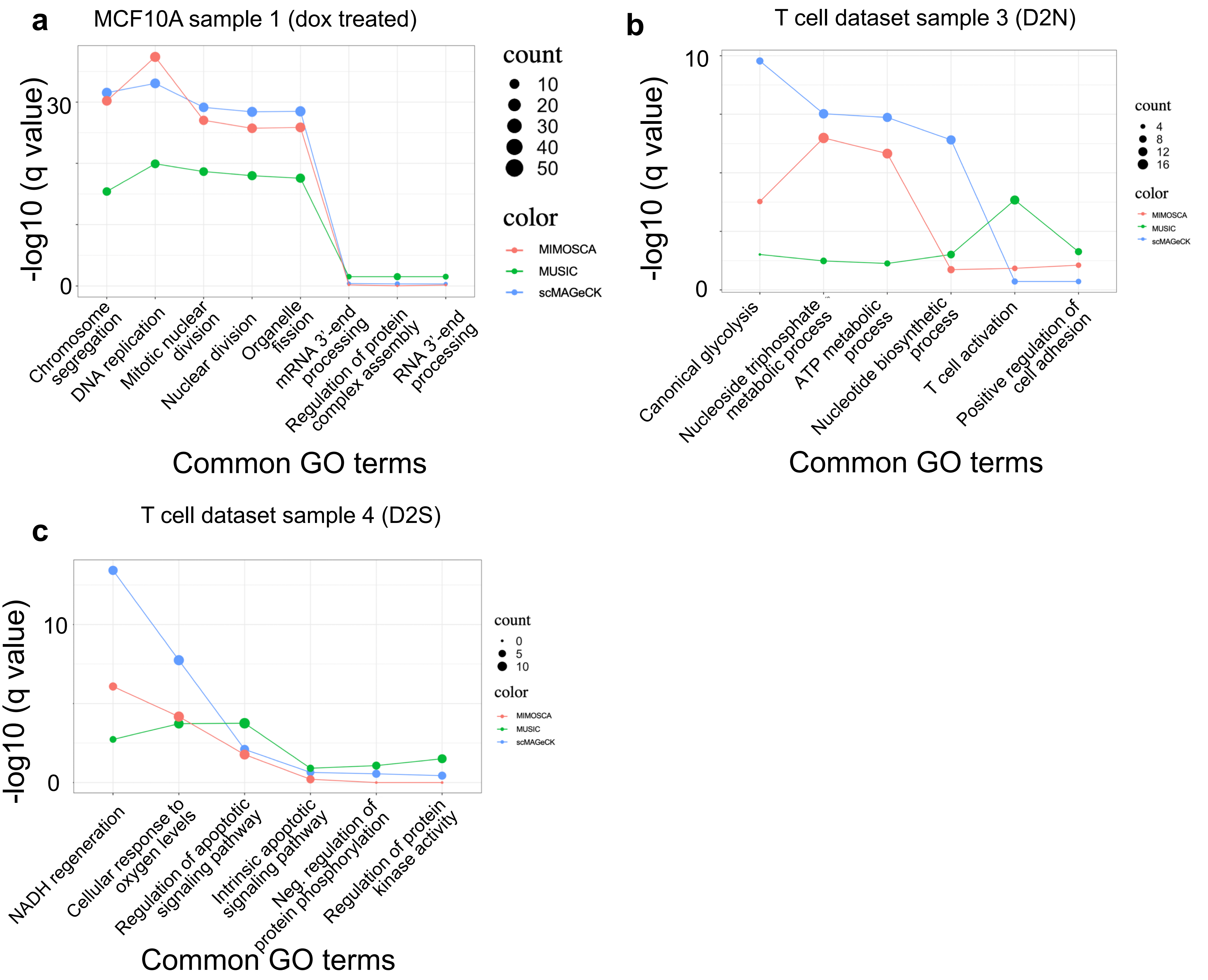

Figure S2: Enriched GO terms and their q values calculated by different methods. GO terms that are identified by at least two out of three methods (with q<0.05) are compared. Among all datasets, three have at least one strong GO terms (q<1e-4) and are shown here (a-c). The rest datasets are shown in Supplementary Figure 3.

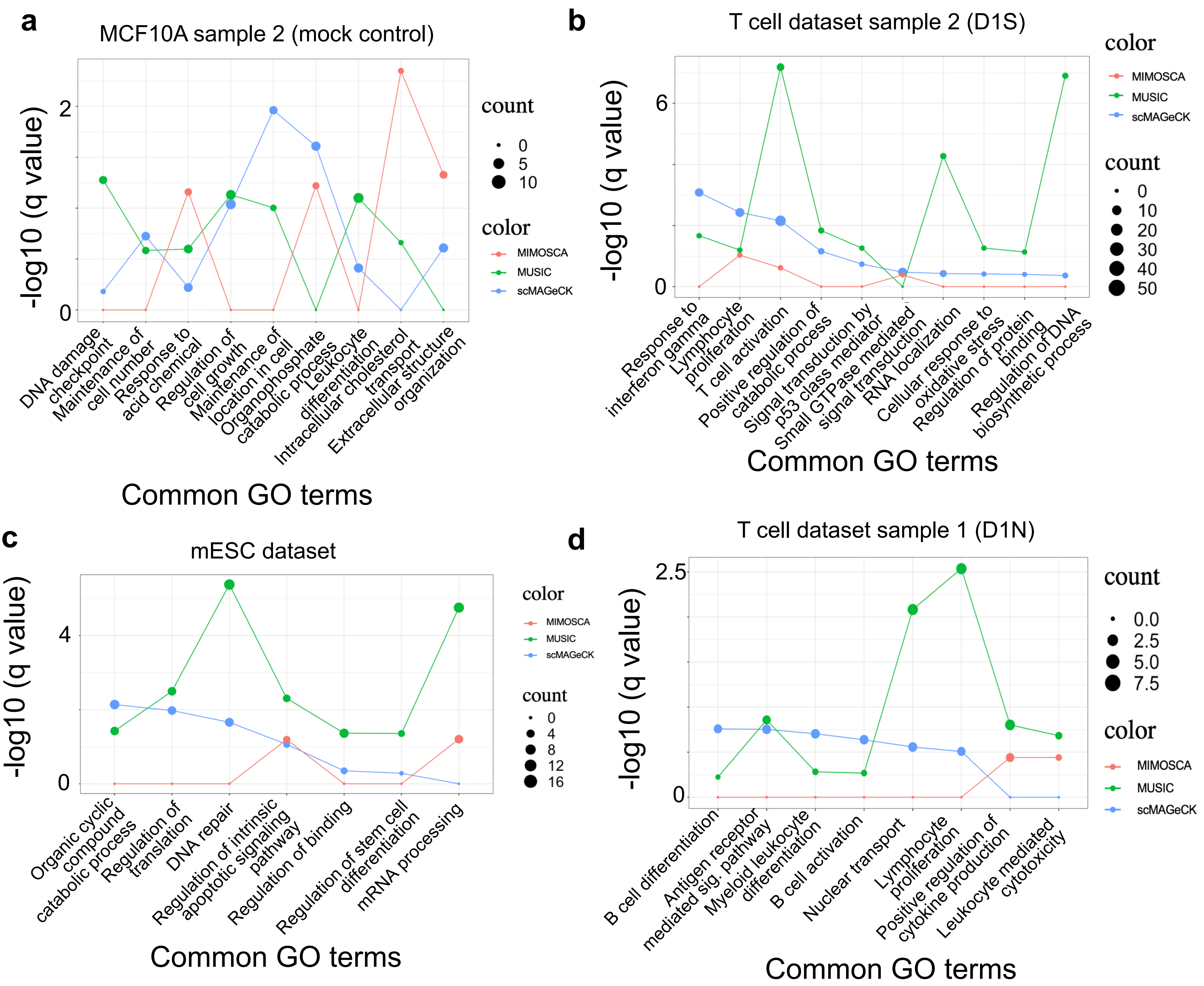

Figure S3: Enriched GO terms and their q values calculated by different methods. GO terms that are identified by at least two out of three methods (with q<0.05) are compared. Among all datasets, three have at least one strong GO terms (q<1e-4) and are shown in Supplementary Figure 2. The rest datasets are shown here.

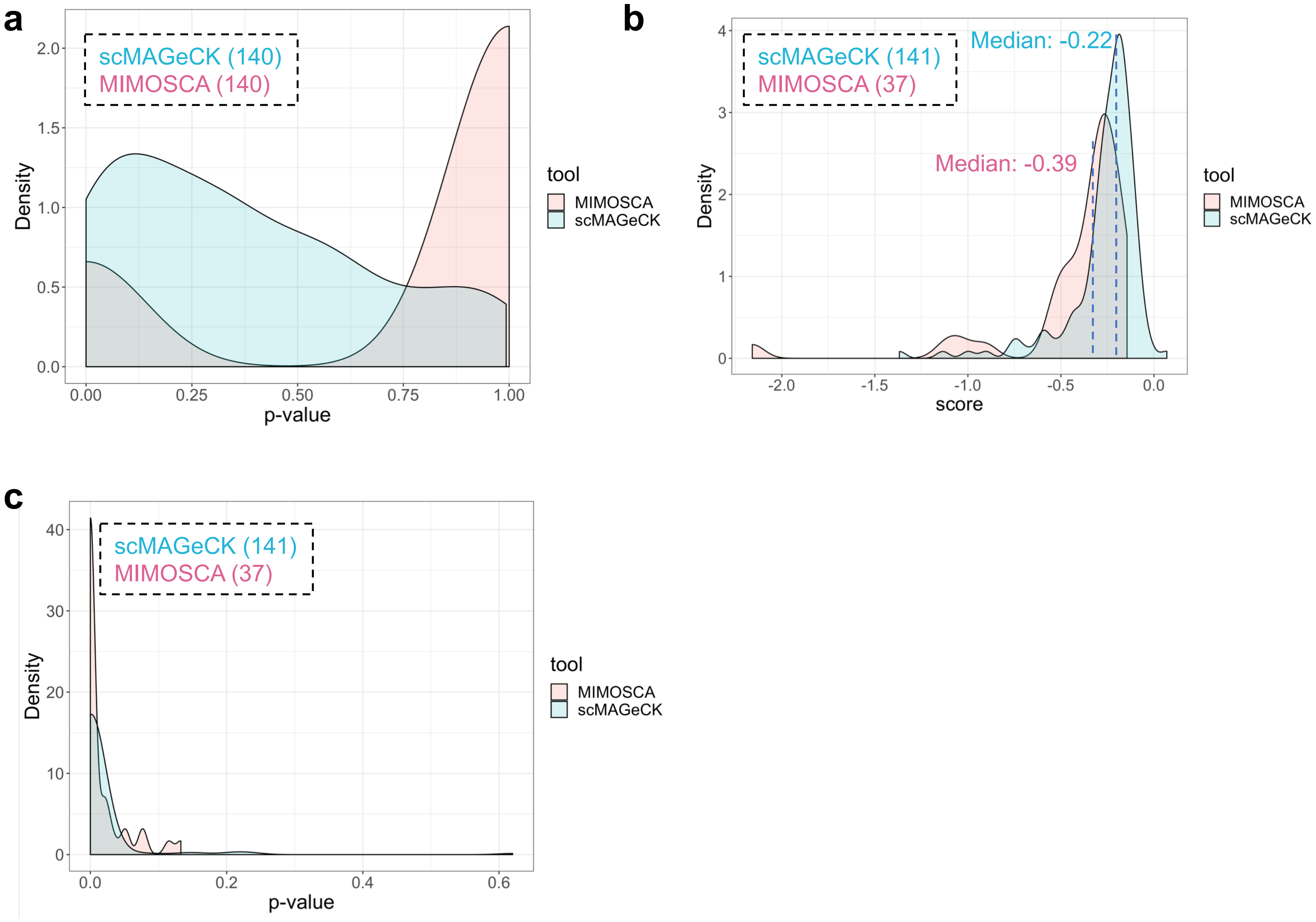

Figure S4: The p value distribution of canonical enhancer-gene pairs in one dataset of K562 (low MOI, a), and the score/p value distribution on another dataset (high MOI, b-c). The number of pairs identified by each method is shown in parenthesis.

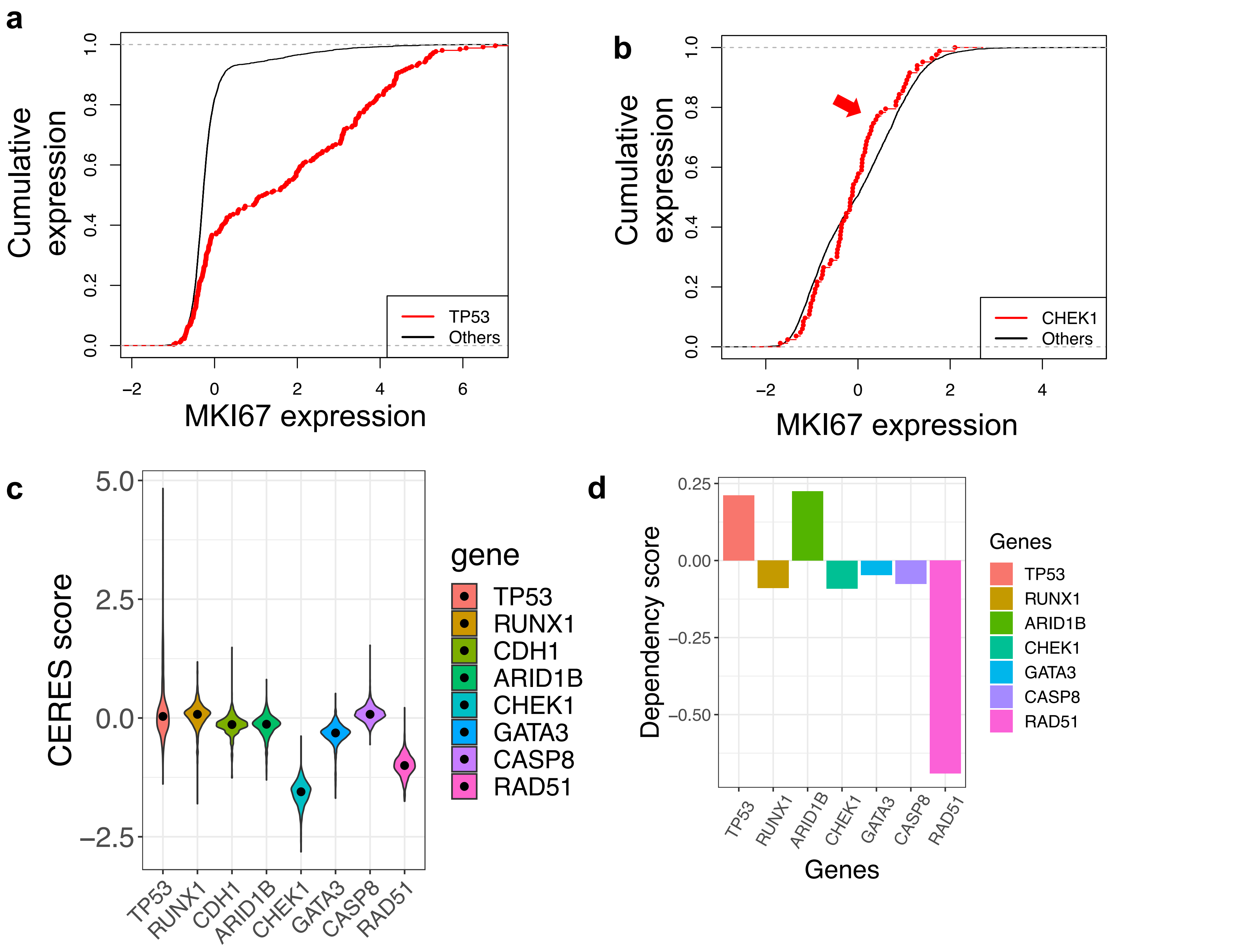

Figure S5: a-b, The cumulative distribution of MKI67 expression upon TP53 knockout (a) and CHEK1 knockout (b). A small fraction of single cells with CHEK1 KO (red arrow) has an decreased MKI67 expression, confirming the results in Fig. 2b. c, The CERES score of top hits in Fig. 2a-b in the 500 cell line CRISPR screening data in DepMap. d, The dependency scores of top hits in Fig. 2a-b in MCF10A RNAi screens in DepMap. CDH1 was not screened in RNAi screening dataset.

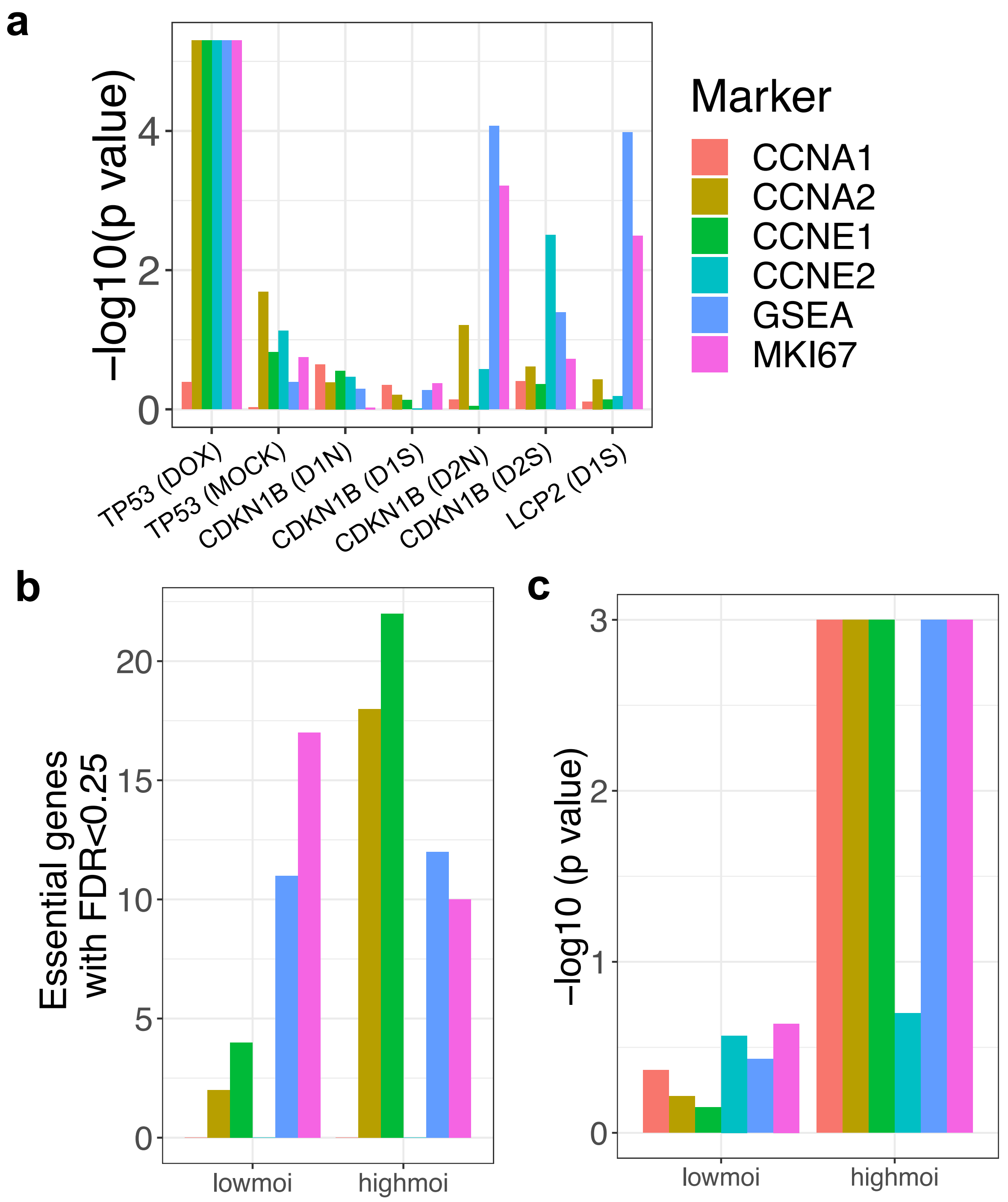

Figure S6: The effect of different proliferation markers (or marker combinations). a, The scMAGeCK-RRA p value of known (or validated) markers that suppress (TP53, CDKN1B) or induce proliferation (LCP2). A gene signature from GSEA database is tested as well (marked as "GSEA", blue bar). b, The number of k562 essential genes that whose knockdown reduces proliferation markers with statistical significance (FDR<0.25). 53 essential genes are tested that are considered essential (using K562 CRISPR screening data) and whose TSS are targeted in K562 dataset. c, The enrichment of 53 essential genes in (b) among genes/enhancers that are negatively selected using different markers. Gene-Set Enrichment Analysis (GSEA) was used to calculate the p values.

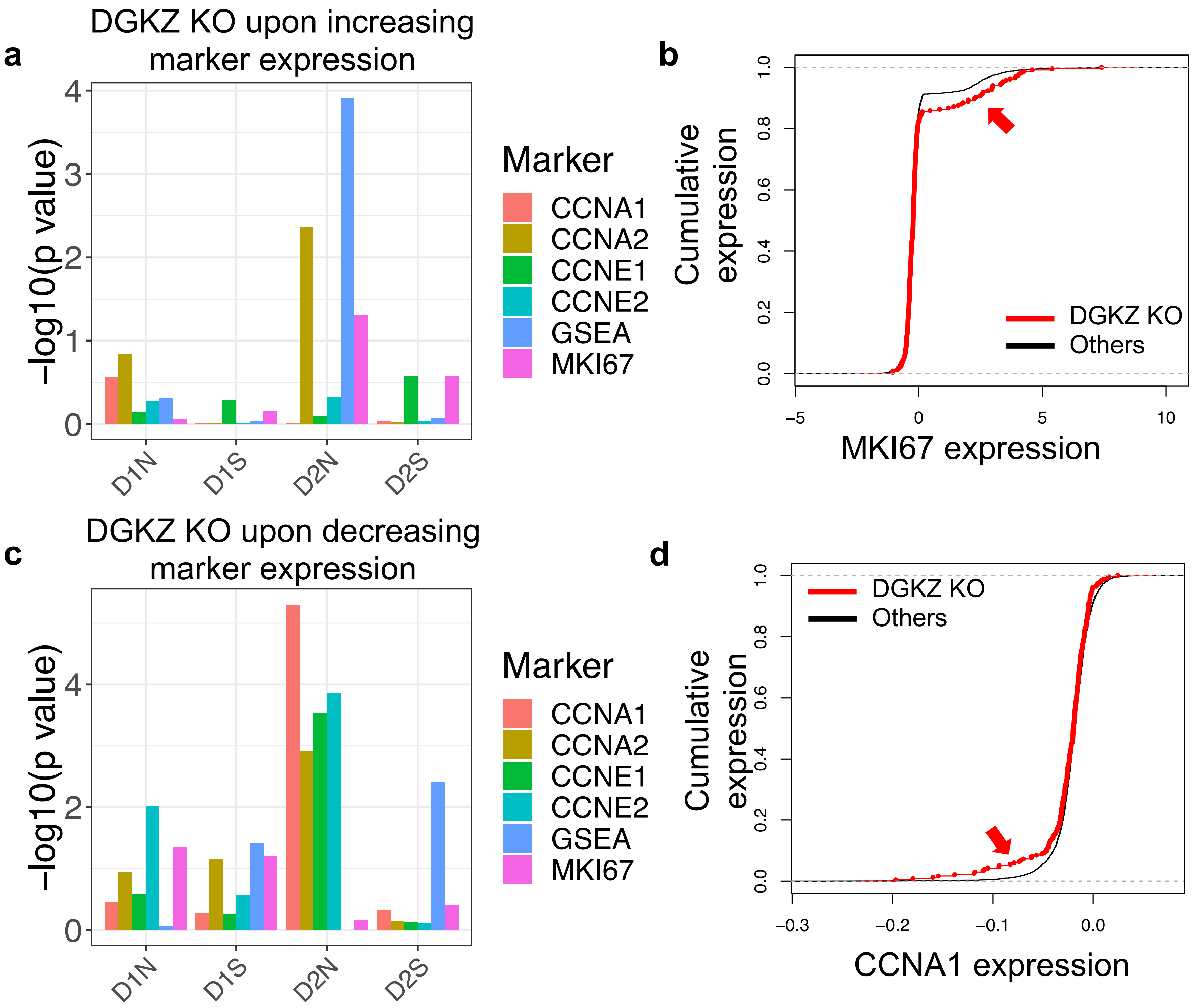

Figure S7: The effect of DGKZ knockout on different proliferation markers. a, The KO effect on increasing marker expression. b, A small fraction of single cells with DGKZ KO (red arrow) has an increased MKI67 expression, confirming the results in a. c-d, DGKZ KO decreases several cyclin gene expressions (c), and is confirmed by cumulative expression plot (d).

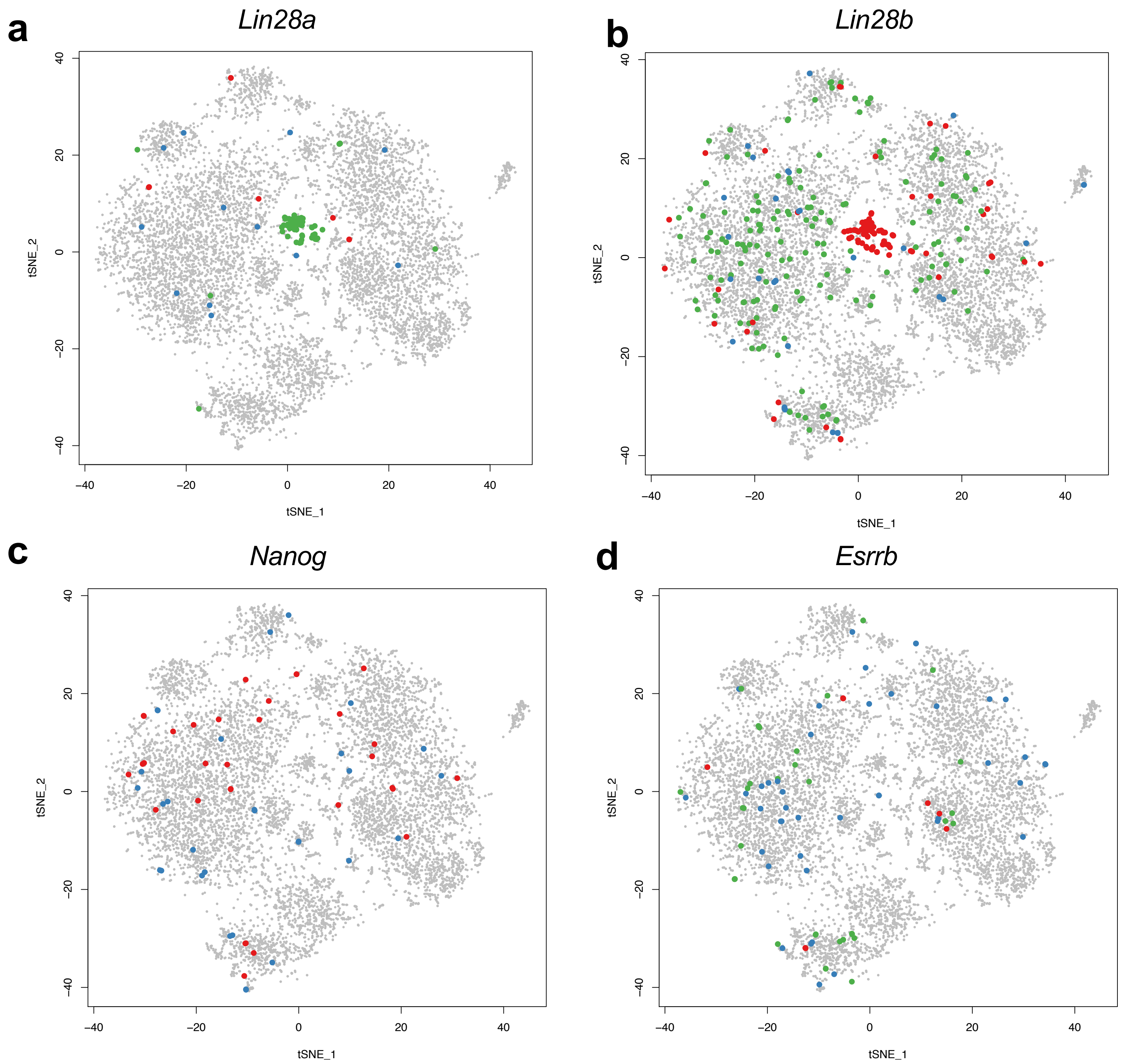

Figure S8: t-SNE clustering of all single cells in mESC dataset. Genes that are enriched in clusters, including *Lin28a-b* (a-b), and representative genes not enriched in any clusters, including *Nanog* and *Esrrb* (c-d), are shown. Dots with different colors represent different sgRNAs targeting that gene.

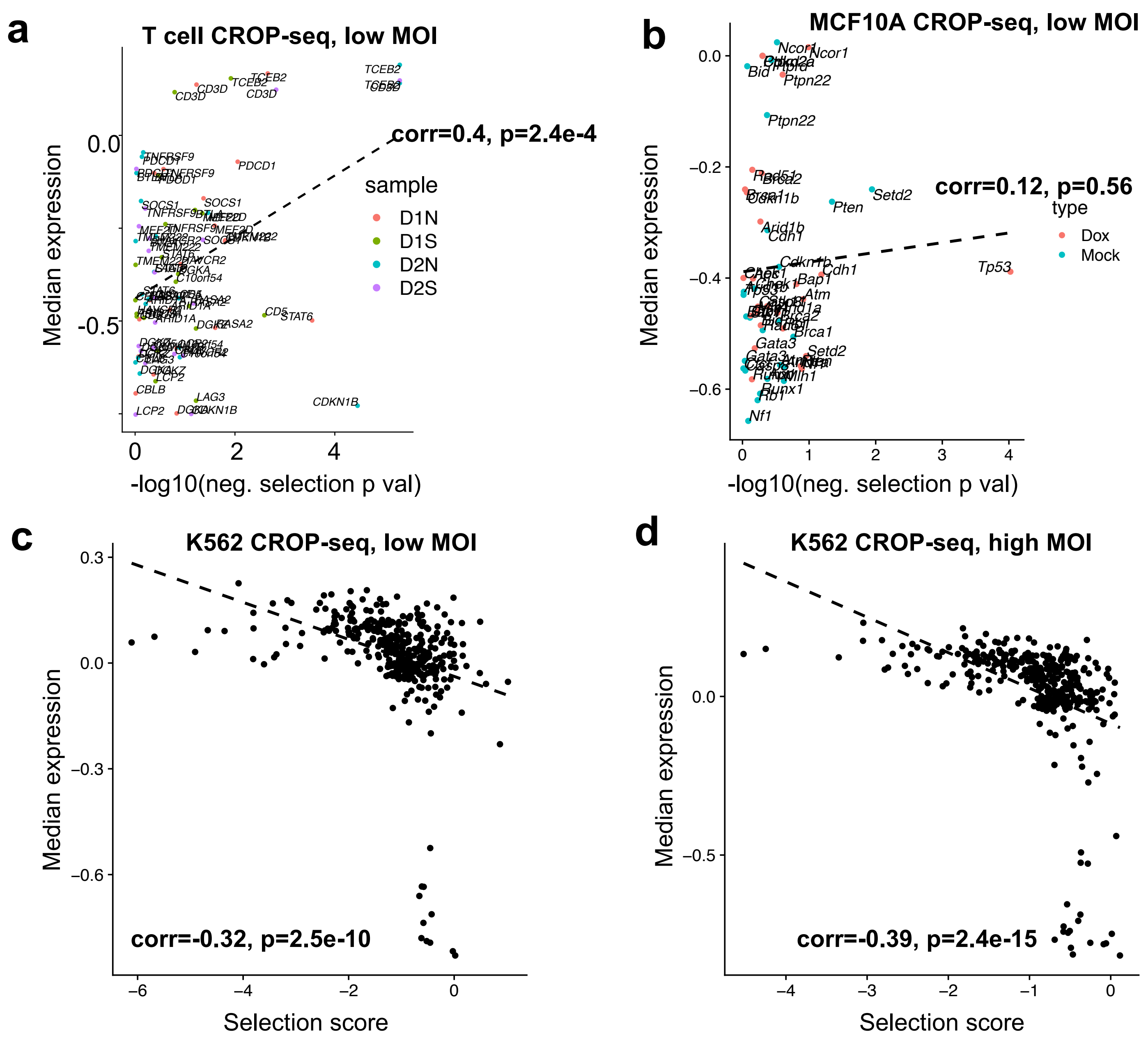

Figure S9: The knockout effect on target gene down-regulation (measured by negative selection p value or selection score) and the average target expression in CROP-seq datasets. Different samples are marked in different colors.

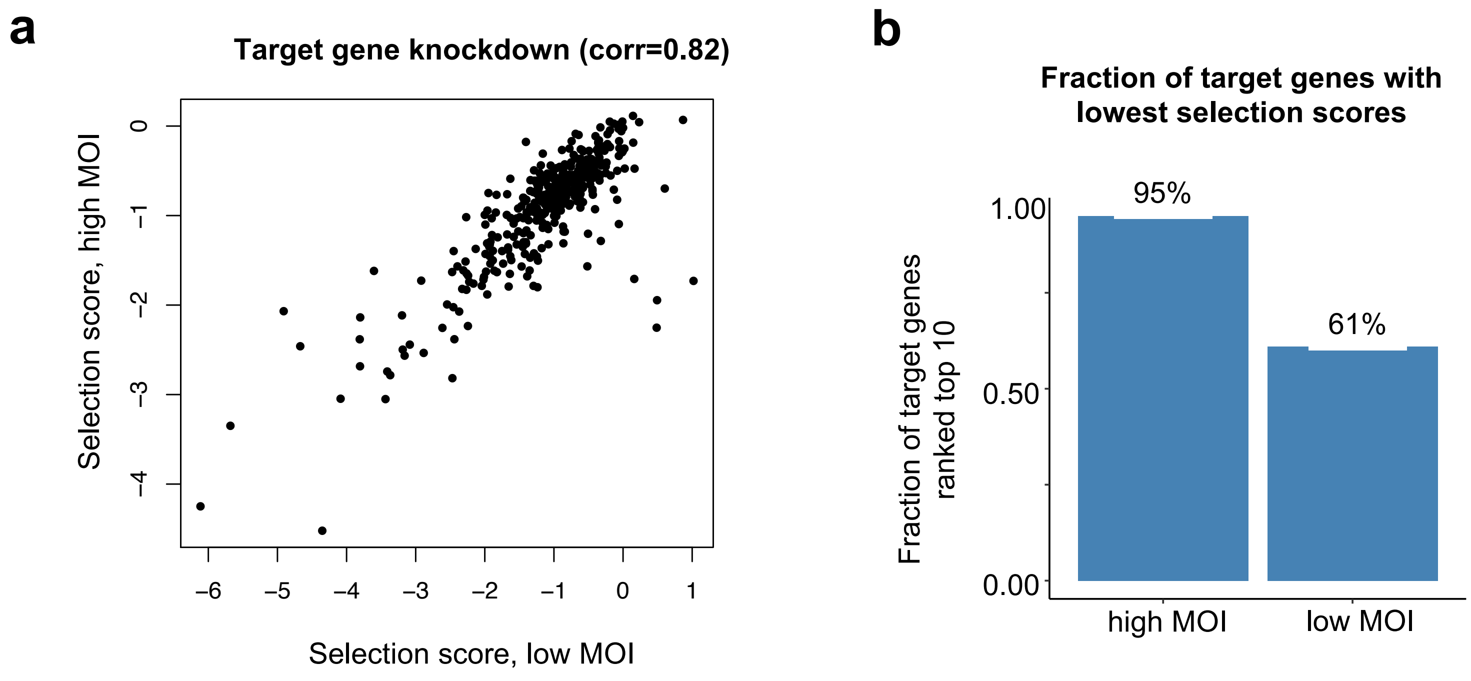

Figure S10. Target gene knockdown in K562 CROP-seq. a, The correlation of selection score in high/low MOI screens. b, Fraction of genes that have the lowest scores (ranked top 10) across all possible genes in high/low MOI screens.

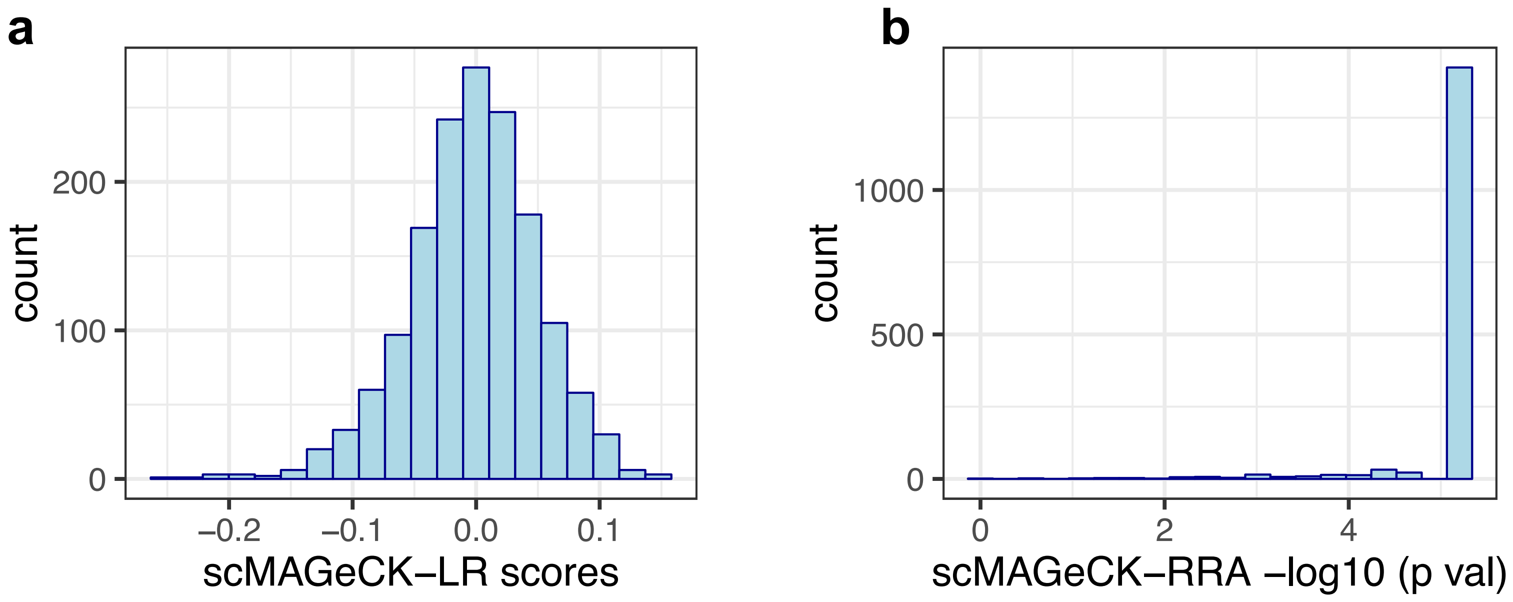

Figure S11. The distribution of LR scores (a) and RRA log p values (b) on K562 high MOI data. scMAGeCK-RRA failed in high MOI condition, as it identified almost all of the hits are significant (b).
