# Supplementary material for: scMAGeCK links genotypes with multiple phenotypes in single-cell CRISPR screens
Source: Genome Biol. 2020 Jan 24;21:19. doi: 10.1186/s13059-020-1928-4 (PMC6979386; doi:10.1186/s13059-020-1928-4)
Supplement: Supplementary file 2 — Review history (DOCX 936 kb) [file 13059_2020_1928_MOESM2_ESM.docx]

Review History

**First round of review**

**Reviewer 1**

**Comments to author:**

Yang et al. Linking genotypes with multiple phenotypes in single-cell CRISPR screens


The manuscript "Linking genotypes with multiple phenotypes in single-cell CRISPR screens" describes a computational framework to analyze datasets from emerging technologies such as Perturb-Seq, CRISP-Seq, and CROP-seq. Tools interpreting large datasets from these technologies will be valuable; however, in terms of novelty, this model seems like a modification and application of the original MAGeCK linear regression to analyze a different type of dataset. Additionally, I have several other key questions.

Comments:
1) Although the authors compare the performance of scMAGeCK to clustering analysis, there are other recent models that have investigated scCRISPR screening data (the authors do describe their limitations in the discussion). However, it would still be important to understand how scMAGeCK performs compared to other tools such as: MUSIC (Nature Communication 2019, Duan B et al) or MIMOSCA (Cell 2016, Dixit A et al). Specifically, showing the ranking of the genes compares between MUSIC and scMAGeCK (using MKI67 as a proliferation marker) would be one approach.


2) MKI67 is a widely used marker in cancer histology. Is there a systematic analysis to show that MKI67 expression is the best predictive marker for annotating proliferation? How does it compare with using other expressions like cyclin A or E? Will using proliferation gene expression signature (i.e. GSEA gene lists) potentially improve the model than relying on expression of one gene as expression of MKI67 might drastically differ between different stages of the cell cycle would drastically alter the data at a single-cell level?  In addition, using more than one expression marker of proliferation would likely help demonstrate the robustness of the data analysis.  In other words, one would predict recovering the same relevant genes when using a highly correlated second or third marker of proliferation.


3) Similarly, to the point stated above in comment 2, cross-analysis of CRISPR genes analyzed in this dataset (i.e. Fig 2) do not correspond well to the comprehensive datasets like the AVANA CRISPR screen. For examples genes like DGKZ actually decreases growth in over 500 cell lines. Would using other proliferation markers provide different results or is this inherent in the actual screening data?  Is the comparison to functional perturbations in genome-wide approaches (not by single-cell) a mechanism by which one can orthogonally corroborate these data in particular in the MCF10A data set.


4) scCRISPR screening have shown to be noisy and sparse including the published datasets used in this study. In terms of filtration process, are expressions of the gene the sgRNA is intending to knockout used to evaluate/filter the cells for further analysis?

5) minor comment: I am not sure what is meant by saying one can detect the loss of expression of the targeted gene "25-95%" of the time.  This statement implies that there is more than one experimental observation supporting this.  If so, why do the authors not report the mean and/or mean plus error.  Of course, this statement implies a significant issue with noise in your general perturb-seq experiment.

**Reviewer 2**

**Comments to author:**

In this report, Yang et al develop scMAGeCK to analyze scRNAseq with CRISPR screen datasets. The authors extend their previous MAGeCK analysis tools used for pooled CRISPR screens. Overall this is a very helpful analysis method which should be broadly useful as scRNAseq CRISPR screens are used more frequently. There are no widely used existing tools for this type of experiment, so this could become the new standard in the area. My comments relate to clarification of a few issues, such as handling dropouts, comparison of RRA vs LR tools, network analysis.

Some comments:
1.     Authors exclude dropouts, at least for RRA tool. This may help to avoid low quality cells, but may lead to information loss. Some dropouts may be due to technical limitation of scRNA-seq. However other dropouts could be due to real perturbation with reduced expression of gene. Perhaps filtering out dropouts might exacerbate the challenge of detecting effects for lowly expressed genes. Are there strategies that could keep the dropouts? At least authors could show the effect of including vs excluding dropouts.
2.     How was CD3D selected as an example? Is this the exception or rule? What about near hits for CD3D knockout, like TMEM222, MEF2D, LAG3 as shown in 1c? It would be helpful to see where these fall in the clusters in 1d-e.
3.     It would be helpful if the authors could explore more the failures of scMAGeCK-RRA to find target genes. In the MCF10A dataset (Fig 1b) most target genes were not detected. In T cell dataset (Fig 1b), many target genes were not detected. It would be helpful to show illustrative examples to understand failures. Is this due to low guide KO efficiency, low expression of target gene (many dropouts), or other problems?
4.     Why does the K562 dataset perform so much better in terms of few failures to detect target genes? Is this the low MOI or high MOI K562 data? (If high MOI was LR used instead of RRA?) Exploring what determines success vs failure would be helpful not only to optimize analysis but to suggest experimental parameters to achieve high quality interpretable datasets.
5.     Even though not enriched in clusters, are the targeted cells in Fig 1d randomly or nonrandomly distributed across tSNE plots? If nonrandom perhaps another method could detect enrichment even if not standard clustering. (Also figure or legend should explain difference between red and blue colored symbols.)
6.     What is justification for using many different FDR thresholds throughout the manuscript (0.01, 0.1. 0.15, 0.3, etc)? Why do some figures show p-value and others adjusted p-value? Maybe just show FDR as axes and not p-value for consistency and simplicity? It would be helpful if the authors provided guidance on how to select an appropriate FDR for a given dataset.
7.     Are the genome-wide CRISPR screens in Fig 2d-e previously published or newly performed? This should be clarified. Also need to clarify if these were conducted under "stimulating", "non-stimulating" or some other condition (as compared to Fig 2c)?
8.     Why is RRA not suitable under high MOI conditions? Since more and more screens will use high MOI, this will be a limitation for the RRA method. I might think that the distribution of the other guides would be similar in the cells with and without the target guide, so that the RRA method might still work. It would be helpful to show examples of how RRA fails under high MOI (even in supplement).
9.     Reciprocally, it would be helpful to show why is RRA necessary if LR can overcome the problem of high MOI? What would the results from Fig 1b (target gene knockout) look like using LR instead of RRA tool?
10.     In Fig 3b, why is RASA2 not shown as a negative regulator of proliferation under "stimulating" conditions as described in the text? This should be clarified.
11.     Could the authors comment on the performance of Lin28a and Lin28b in their CROP-seq experiment? Both genes were not validated as knocking out their target by RRA analysis. Yet each shows a specific clustering pattern in Supp Fig 1. Does this mean that knocking out these genes leads to a strong effect on a rare subset of cells?
12.     Again it would be helpful for authors to try to understand what determined the successes vs failures to detect target genes in Fig 4c. The failure rate seems quite high. Would more guides per target gene help? Showing the expression vs selection score for the ES cell data generated by authors could be informative.
13.     Fig 4e. What are the inputs and outputs for the network analysis? Would it be feasible to input all 15 target genes and discover all significant relationships with marker genes? This would seem the most "agnostic" way to look at gene networks.
14.     How do the results of the network analysis change as the number of marker genes increases? E.g. sometimes examples are shown with one marker per phenotype, but other times with multiple markers per phenotype. It is unclear why different numbers of markers per phenotype are used, and how a user should select marker genes. Also how many phenotypes to select?
15.     It is helpful that the authors show the benefit of high MOI. It would also be helpful if the authors could provide some discussion on hypotheses as to why high MOI helps? Is there any tradeoff to high MOI? How to pick best MOI for prospective experiments?
16.     It is helpful that the authors show the challenge of lowly expressed genes. Any strategies to deal with low expressed genes?
17.     In the Results section "scMAGeCK method overview" 3rd paragraph, 3rd line, should be "LR" not "RRA".

**Reviewer 2**

**Comments to author:**

In this manuscript the authors develop a new single-cell CRISPR screening analysis pipeline aka scMAGeCK. They validate this pipeline across multiple experiments: one, identifying genes and enhancers that modulate expression of MKI67 (Ki-67); two, a single-cell CRISPR screen on mouse embryonic stem cells (mESC) to identify genes associated with pluripotency states; and three, exploring key factors that improve the statistical power of single-cell CRISPR screens. Overall the study is comprehensive and well executed. However, as multiple computational pipelines exist for analyzing perturb-seq like screens, the authors must benchmark their tools efficacy vis-a-vis these especially the Regev et al and Weissman et al analysis workflows (similar to table 1 of: https://genomebiology.biomedcentral.com/articles/10.1186/s13059-014-0554-4). A side-by-side comparison would enable broader adoption of the scMAGeCK pipeline.

**Authors Response**

**Point-by-point responses to the reviewers’ comments:**

Reviewer #1: Yang et al. Linking genotypes with multiple phenotypes in single-cell CRISPR screens The manuscript "Linking genotypes with multiple phenotypes in single-cell CRISPR screens" describes a computational framework to analyze datasets from emerging technologies such as Perturb-Seq, CRISP-Seq, and CROP-seq. Tools interpreting large datasets from these technologies will be valuable; however, in terms of novelty, this model seems like a modification and application of the original MAGeCK linear regression to analyze a different type of dataset. Additionally, I have several other key questions.

*Response: We appreciate the reviewer’s positive comment of scMAGeCK to interpret the datasets from emerging single-cell CRISPR screens. From an algorithmic perspective, scMAGeCK is similar with the original MAGeCK RRA and MLE algorithm. However, the novelty mainly lies in applying MAGeCK model to a new data type (single-cell CRISPR screens), which is becoming an important technology to study large-scale gene-gene and enhancer-gene regulations. The novelty of this manuscript also comes from (1) a new algorithm that provides a variety of features to address different needs (see Table 1) and reaches better performance over existing methods (see the “Comparisons with clustering analysis and other algorithms” section); (2) the application of this single-cell CRISPR screen (with scMAGeCK) in identifying regulators of mouse embryonic stem cell differentiation; and (3) the discovery of factors that determine the success of single-cell CRISPR screens, providing guidelines for future experiment designs.*

Comments:

1) Although the authors compare the performance of scMAGeCK to clustering analysis, there are other recent models that have investigated scCRISPR screening data (the authors do describe their limitations in the discussion). However, it would still be important to understand how scMAGeCK performs compared to other tools such as: MUSIC (Nature Communication 2019, Duan B et al) or MIMOSCA (Cell 2016, Dixit A et al). Specifically, showing the ranking of the genes compares between MUSIC and scMAGeCK (using MKI67 as a proliferation marker) would be one approach.

*Response: We systematically compared scMAGeCK with MUSIC and MIMOSCA in the updated section “Comparisons with clustering analysis and other algorithms”. Briefly, we compared the false positive rates of different methods in the permutated CROP-seq datasets (where sgRNA – single cell correspondence is shuffled), the enriched Gene Ontology (GO) terms associated with each perturbed gene, and the sensitivity to detect canonical enhancergene regulations. The comparisons are demonstrated in the new Figure 1d-f and Supplementary Figures 2-4 and are summarized below.*

*First, since MUSIC is an unsupervised method to identify the biological functions of perturbed genes, we systematically compared the enriched Gene Ontology (GO) terms associated with each perturbation. In the permuted CROP-seq dataset, sgRNA labels of single cells are randomly shuffled and any significant term is considered as false positive. Among those, scMAGeCK-LR and MIMOSCA identified fewer enriched GO terms than MUSIC (Fig. 1d). scMAGeCK-LR has the fewest terms in six out of seven CROP-seq samples, demonstrating its good control of false positives. scMAGeCK-RRA was not included as it requires a specific expression marker as input.*

*Second, to evaluate the sensitivity of three methods, we compared the enriched GO terms on the original CROP-seq datasets. Only terms that are found in at least two out of three methods are considered as “ground truth” terms. Among the datasets where at least one strong GO term was found, scMAGeCK achieved better enrichment than other methods, evidenced by its lower q values (Supplementary Fig. 2).*

*Third, to compare the false positive rate of using certain expression markers, we randomly selected expression markers (from protein-coding genes) in permutated CROP-seq datasets, and identified statistically significant genes (FDR<0.05) as a measurement of false positive. Both scMAGeCK modules demonstrated fewer levels of false positives than MIMOSCA, while scMAGeCK-RRA has the fewest number of significant genes as false positives.*

*Last but not least, the original K562 CROP-seq study used an independent approach to identify 145 canonical enhancer-gene pairs (Gasperini et al 2019). Compared with scMAGeCK that outputs almost all of the canonical pairs, MIMOSCA fails to report many of the enhancer-gene pairs (Supplementary fig. 4b) or generates zero scores (Fig. 1f). Collectively, these comparisons demonstrated the good control of false positives and better sensitivity of scMAGeCK over other methods.*

*In addition (also per the request of Reviewer #3), we compared different features of different methods in the new Table 1. Both scMAGeCK modules have different applications and their combination enables users to adapt to different types of single-cell CRISPR screening data.*

*Note that we were not able to compare scMAGeCK with MUSIC in terms of MKI67, since MUSIC is an unsupervised approach and does not take certain expression marker as input.*

2) MKI67 is a widely used marker in cancer histology. Is there a systematic analysis to show that MKI67 expression is the best predictive marker for annotating proliferation? How does it compare with using other expressions like cyclin A or E? Will using proliferation gene expression signature (i.e. GSEA gene lists) potentially improve the model than relying on expression of one gene as expression of MKI67 might drastically differ between different stages of the cell cycle would drastically alter the data at a single-cell level? In addition, using more than one expression marker of proliferation would likely help demonstrate the robustness of the data analysis. In other words, one would predict recovering the same relevant genes when using a highly correlated second or third marker of proliferation.

*Response: We thank the reviewer for the critical comment. To investigate the effect of using different proliferation markers, we compared MKI67, cyclin A (CCNA1/2) and cyclin E (CCNE1/2) in Reviewer-Only Figure 1 and in the new Supplementary Figure 6. We also included one cell-cycle gene signature from GSEA gene list (pathway name: WHITFIELD_CELL_CYCLE_LITERATURE). For MCF10A and T cell dataset, we tested several known or validated gene targets (TP53 in MCF10A dataset, CDKN1B and LCP2 in T cell dataset, respectively), and compared the associated p values in Reviewer-Only Fig 1a. For K562 dataset, we first identified 53 genes in the screening library that are considered as essential, using K562 CRISPR screening data from DepMap. We then calculated how many of these genes negatively regulate proliferation markers with statistical significance (Review-Only Fig. 1b), and whether these essential genes are enriched in the negative selection ranked list using GSEA algorithm (Reviewer-Only Fig. 1c).*

*Different markers may lead to different results in Reviewer-Only Figure 1. Many markers may work well in some datasets (e.g., doxorubicin treated MCF10A and high-MOI K562), while in some T cell datasets, none of the markers have strong p values. Overall, MKI67 (pink bar) and gene signatures (GSEA, blue bar) have better performance compared with cyclin genes.*

*As is pointed out by the reviewer, gene signatures may lead to more robust results since it uses more than one marker. On the other hand, using MKI67 as a single marker has similar performance as gene signature, as MKI67 has a pretty high expression in proliferating cells. Based on the reviewer’s comments and suggestions, we added the following result at the end of the section “Identification of known oncogenic and tumor suppressor genes and enhancers”:*

*“MKI67 is a widely used marker for proliferation. To investigate the effect of different proliferation markers (or marker combinations), we systematically compared MKI67 with cyclin a (CCNA1/2) and cyclin E (CCNE1/2), two cyclin family members that regulate the cell cycle. In addition, we included one cell cycle related gene signature from GSEA MSigDB database (pathway name: WHITFIELD_CELL_CYCLE_LITERATURE) (Subramanian et al., 2005). We tested whether these markers are indicative of known genes (or validated genes) that regulate proliferation (e.g., TP53, CDKN1B, LCP2; Supplementary Fig. 6a). For K562 dataset, 53 essential genes were identified from K562 CRISPR screening (Meyers et al., 2017) whose TSS are targeted in the CROP-seq library. These gene are evaluated whether their knockdowns reduced proliferation marker expressions (Supplementary Fig. 6b), and their enrichment among all genes/enhancers in the library using GSEA (Supplementary Fig. 6c). Overall, gene signatures and MKI67 worked better than cyclins to identify known or validated proliferation-associated genes (with the only exception in K562 high MOI where more essential genes are identified from CCNA2/CCNE1). In contrast, the behaviors of cyclin genes vary across datasets: some cyclin genes work equally well or better than MKI67/signature (e.g., CCNE1 in high-MOI K562), but none is served as a stable indicator of proliferation. Interestingly, some markers provide opposite directions in certain perturbations (e.g., DGKZ knockout; Supplementary Fig. 7). These results indicate that various qualities of the datasets, compositions of cells at different stages, and the use of different markers may contribute to different aspects upon one single phenotype of cell proliferation.”*

3) Similarly, to the point stated above in comment 2, cross-analysis of CRISPR genes analyzed in this dataset (i.e. Fig 2) do not correspond well to the comprehensive datasets like the AVANA CRISPR screen. For examples genes like DGKZ actually decreases growth in over 500 cell lines. Would using other proliferation markers provide different results or is this inherent in the actual screening data? Is the comparison to functional perturbations in genome-wide approaches (not by single-cell) a mechanism by which one can orthogonally corroborate these data in particular in the MCF10A data set.

*Response: We tested different proliferation markers mentioned in comment #2 upon DGKZ knockout (Reviewer-Only Fig. 2, also in the new Supplementary Fig. 7). DGKZ knockout increases MKI67 and cell cycle signature expression. This is consistent with the genome-wide CRISPR screening data in Shifrut et al. 2018 (also presented in Fig. 2e), where DGKZ is ranked top in suppressing T cell proliferation. Interestingly, DGKZ knockout decreases all cyclin expressions, consistent with its role in AVANA CRISPR screen mentioned by the reviewer. We confirmed both cases are true in the CROP-seq data (see red arrows in Reviewer-Only Fig. 2b,d). This is consistent with our finding in response to comment #2 that different proliferation markers may lead to different results. One possible reason is that primary human T cell proliferation phenotype may be different from cancer cell lines growth, and these cyclin genes may serve as a marker of different T cell status rather than proliferation.*

*As is suggested by the reviewer, we also compared AVANA CRISPR screening results of the top hits in MCF10A CROP-seq (Fig. 2a-b): TP53, RUNX1, ARID1B, putative tumor suppressor genes; and CDH1, CHEK1, GATA3, CASP8, and RAD51, putative oncogenes. The CERES scores of these 8 genes across 500 cell lines are shown in Reviewer-Only Figure 3 (also in the new Supplementary Fig. 5c). Note that AVANA CRISPR screening dataset does not include MCF10A cells.*

**Reviewer-Only Figure 3 (also in Supplementary Fig. 5c).** The CERES score of selected genes in the 500 cell line screening data in DepMap.

*On average, TP53 and RUNX1 have positive CERES scores while CHEK1, GATA3 and RAD51 have negative CERES scores, consistent with the CROP-seq experiments. Interestingly, CDH1, ARID1B and CASP8 appear to have opposite directions compared with CROP-seq results. This may due to the cell type-specific roles of these genes. For example, ARID1B as a negative CERES score distribution, but ARID1B is generally considered a tumor suppressor gene (e.g., Khursheed et al. Br J Cancer 2013). We incorporated these results into Supplementary Fig. 5c, also discussed it in the revised manuscript:*

*“Many of these genes are consistent with their roles as tumor suppressors or oncogenes in genome-wide CRISPR screens (Supplementary Fig. 5c). Some genes have opposite compared with genome-wide CRISPR screens, an indication that they may function as a cancer type-specific manner.”*

4) scCRISPR screening have shown to be noisy and sparse including the published datasets used in this study. In terms of filtration process, are expressions of the gene the sgRNA is intending to knockout used to evaluate/filter the cells for further analysis?

*Response: The expressions of target genes are not used to filter the cells. Instead, we evaluated the target gene expression as a factor that determines the knockout efficiency of scCRISPR screens (Fig. 5a-d).*

5) minor comment: I am not sure what is meant by saying one can detect the loss of expression of the targeted gene "25-95%" of the time. This statement implies that there is more than one experimental observation supporting this. If so, why do the authors not report the mean and/or mean plus error. Of course, this statement implies a significant issue with noise in your general perturb-seq experiment.

*Response: We apologize for the confusion. The “25%-95% rate is based on all the datasets in Fig. 1b, not on one particular dataset. We revised our statement as follows (2nd paragraph of the section “Comparisons with clustering analysis and other algorithms”):*

*“In two out of three datasets, we found that 25% (MCF10A data) and 95% (T cell data) of the target genes have reduced expressions with statistical significance, respectively, a demonstration that scMAGeCK-RRA better captures the effect of gene perturbation than the clustering analysis.”*

Reviewer #2: In this report, Yang et al develop scMAGeCK to analyze scRNAseq with CRISPR screen datasets. The authors extend their previous MAGeCK analysis tools used for pooled CRISPR screens. Overall this is a very helpful analysis method which should be broadly useful as scRNAseq CRISPR screens are used more frequently. There are no widely used existing tools for this type of experiment, so this could become the new standard in the area. My comments relate to clarification of a few issues, such as handling dropouts, comparison of RRA vs LR tools, network analysis.

Some comments:

1. Authors exclude dropouts, at least for RRA tool. This may help to avoid low quality cells, but may lead to information loss. Some dropouts may be due to technical limitation of scRNAseq. However other dropouts could be due to real perturbation with reduced expression of gene. Perhaps filtering out dropouts might exacerbate the challenge of detecting effects for lowly expressed genes. Are there strategies that could keep the dropouts? At least authors could show the effect of including vs excluding dropouts.

*Response: Cells with dropouts are not excluded in the RRA algorithm. Instead, cells with dropout expressions are still in the ranked list, but are excluded when RRA tries to find a cell with minimum p value (i.e. strongest enrichment). This is because cells with dropouts have identical rankings that are not handled well in RRA. We apologize for the confusion, and revised the results and methods part accordingly. More specifically, we changed the sentences in the “scMAGeCK method overview” section as follows:*

*“The alpha parameter is set to limit RRA on single cells whose marker expression is greater than zero, therefore minimizing the effect of possible dropout events.”*

*We also changed the “scMAGeCK algorithm” part in the Methods section as follows:*

*“For positive selection (cells with gene X knockout are enriched in higher A expression), the significance score of the gene, the ρ value, is defined as ρ = min(p1, p2, …, pj), where j out of the n single-cells targeting gene X have non-zero read count on gene A. For negative selection, single-cells that are ranked in the front will have zero counts (dropouts). Therefore, we calculated ρ = min(pj+1, pj+2, …, pn) where the first j single cells have zero counts on gene A (and are excluded from the calculation of ρ).”*

*Indeed, as the reviewer pointed out, simply removing dropouts will lose information contained in the droupout cells. For example, in the MCF10A dataset, removing cells with zero MKI67 counts will lose 90% cells used for ranking (only 408 cells remained out of 4063 cells), decreasing the statistical power, as is indicated in Reviewer-Only Figure 4 by an overall increase of the p values.*

2. How was CD3D selected as an example? Is this the exception or rule? What about near hits for CD3D knockout, like TMEM222, MEF2D, LAG3 as shown in 1c? It would be helpful to see where these fall in the clusters in 1d-e.

*Response: CD3D is a randomly selected as an example. Other hits have similar distribution in a t-SNE plot including TMEM222, MEF2D and LAG3. We demonstrated their distributions in Reviewer-Only Figure 5 and in the new Supplementary Figure 1.*

3. It would be helpful if the authors could explore more the failures of scMAGeCK-RRA to find target genes. In the MCF10A dataset (Fig 1b) most target genes were not detected. In T cell dataset (Fig 1b), many target genes were not detected. It would be helpful to show illustrative examples to understand failures. Is this due to low guide KO efficiency, low expression of target gene (many dropouts), or other problems?

*Response: There may be various reasons to the failures presented in Fig. 1b, including the low guide KO efficiency and low target gene expression. As is discussed in the last section (“High target expression and high MOI improves the power of single-cell CRISPR screening”), target gene expression is a possible factor that can explain the failures of some cases (see the original Fig. 5a-c). Low guide KO efficiency is another possible reason, however it’s challenging to evaluate this effect, since there’s no direct measurement on the outcome of each sgRNA using scRNA-seq. Other possible reasons may be (1) target gene knockout may not necessarily reduce target gene expression, and (2) there are not enough single cells for certain gene knockout to reach a statistical significance in scMAGeCK-RRA. (1) is demonstrated in the MCF10A publication (Hill et al. 2018), where TP53 knockout cells change the expressions of TP53 targets (CDKN1A and TP53I3) rather than TP53 itself. (2) is illustrated in Fig. 1b and Fig. 5e-g, where high MOI CROP-seq that includes more cells per knockout (marked “1” in K562 dataset in Fig. 1b) reaches better knockdown effect than low MOI.*

*We added in the discussion section to address the reviewer’s concerns as follows:*

*“Most of the CROP-seq datasets based on target gene knockout have a low successful rate (Fig. 1b). There may be various reasons to the failures, including (1) low target gene expression, (2) low guide knockout efficiency, and (3) not enough single cells to reach a statistical significance. Therefore, to increase the success rate, users may pick up genes with moderate or high expression, and ensure that there are enough number of cells for the analysis (e.g., by increasing MOI). One caveat of this approach is that target gene knockout may not necessarily reduce its expression (Hill et al. 2018). As more CROP-seq (or other types of single-cell technology) datasets accumulate, we may be able to study how guide knockout efficiency affect the outcome of the screen.”*

4. Why does the K562 dataset perform so much better in terms of few failures to detect target genes? Is this the low MOI or high MOI K562 data? (If high MOI was LR used instead of RRA?) Exploring what determines success vs failure would be helpful not only to optimize analysis but to suggest experimental parameters to achieve high quality interpretable datasets.

*Response: Both K562 low and high MOI data performed better than other datasets (Fig. 1b). For high MOI, LR is used instead of RRA. Two possible reasons may contribute to the high successful rate. First, CRISPR inhibition (CRISPRi) was used to directly knock down the expression of target gene expression. In contrast, others use CRISPR-Cas9 to knockout target gene, whose expression may not be affected (as is discussed in response to comment #3). Second, genes selected in the K562 screening generally have high expression in K562 cell line (Gasperini et al. 2019), a factor that we found to increase the success rate (Fig. 5). We updated the last paragraph in the section of “High target expression and high MOI improves the power of single-cell CRISPR screening” as follows:*

*“Across different CROP-seq datasets, K562 performed better than the others (Fig. 1b) as both conditions reached higher numbers of down-regulated genes upon knockdown. Two possible reasons may contribute to the high successful rate. First, CRISPR inhibition (CRISPRi) was used in K562 datasets to directly knock down the expression of target gene expression. In contrast, others datasets use CRISPR-Cas9 to knockout target gene, whose expression may not be affected (Hill et al. 2018). Second, genes selected in the K562 screening generally have high expression in K562 cell line (Gasperini et al. 2019), a factor that contributes to the high success rate (Fig. 5). Collectively, these results indicate that to reach better knockdown effect in CROP-seq experiments, users may select genes with moderate or high expressions, and increase the number of cells per each gene perturbation (by increasing MOI).”*

5. Even though not enriched in clusters, are the targeted cells in Fig 1d randomly or nonrandomly distributed across tSNE plots? If nonrandom perhaps another method could detect enrichment even if not standard clustering. (Also figure or legend should explain difference between red and blue colored symbols.)

*Response: The most common and straightforward approach is still to use chi-squared test or hypergeometric test based on the clustering results (e.g., in Shifrut et al. 2018 and in Gate et al. bioRxiv, 2019). This approach can detect the nonrandom distribution in tSNE plots. The problem of this approach is that (1) it relies on the outcomes of the clustering algorithm, and (2) the enrichment in the cluster is relatively small even though the p values are strong. For example, only two genes have strong enrichment in certain clusters (>30%) with statistical significance (adjusted p value <0.25) in T cell CROP-seq (Reviewer-only Fig. 6, also in Supplementary Fig. 1f), as is mentioned in the manuscript (Fig. 1b). Most of the significant results have weak enrichment (0-15%), making it challenging it interpret the biological meanings. We included this result into out revision (Supplementary Fig. 1f).*

*The different colors in Fig. 1d (now in the new Supplementary Fig. 1a) represent different sgRNAs. We revised the figure legend accordingly.*

6. What is justification for using many different FDR thresholds throughout the manuscript (0.01, 0.1. 0.15, 0.3, etc)? Why do some figures show p-value and others adjusted p-value? Maybe just show FDR as axes and not p-value for consistency and simplicity? It would be helpful if the authors provided guidance on how to select an appropriate FDR for a given dataset.

*Response: Different datasets may have various qualities that affect the FDR calculation. Therefore, we use different FDR thresholds in analyzing different datasets. Throughout the manuscript, we tried to employ stringent FDR cutoffs (e.g., 0.01 in Fig. 2d-e, 0.05 in Fig. 4d). For MCF10A dataset, the threshold is higher (0.15 in Fig. 2a, and 0.3 in Fig. 2b), mainly because of the relatively low quality of the data (see Fig. 1b), and the top hits can still be validated from literature and other data source including DepMap (Reviewer-Only Fig. 3).*

*Per reviewer’s suggestions, we changed the axes from p-value to FDR in Fig. 2a,b,d,e,f,h,i, and Fig. 5b. We didn’t change Fig. 5a and Fig. 5f, since Fig. 5a is used to evaluate the association between raw p values with gene expressions, and we only looked at the effect of CD46 gene expression upon CD46 knockdown (red dot). In both cases, multiple comparison issues are not involved.*

*The choice of appropriate FDR depends on how stringent the users would like the results would be. Users may select low thresholds if they want fewer but more reliable results, and high thresholds if they want more results and can tolerate high false positives. We discussed this issue at the end of the discussion section as follows:*

*“As the quality of different CROP-seq datasets varies (e.g., Fig. 1b), choosing a proper false discovery rate (FDR) cutoff is an essential step. The choice of appropriate FDR depends on how stringent the users would like the results would be. Users may select low thresholds (e.g., 0.01) if they want fewer but more reliable results, and high thresholds (e.g., 0.25) if more results are needed and a high false positive rate can be tolerated.”*

7. Are the genome-wide CRISPR screens in Fig 2d-e previously published or newly performed? This should be clarified. Also need to clarify if these were conducted under "stimulating", "non-stimulating" or some other condition (as compared to Fig 2c)?

*Response: The genome-wide CRISPR screens are previously published in Shifrut et al. 2018, in stimulating T cells. We clarified this point in the manuscript and in the figure.*

8. Why is RRA not suitable under high MOI conditions? Since more and more screens will use high MOI, this will be a limitation for the RRA method. I might think that the distribution of the other guides would be similar in the cells with and without the target guide, so that the RRA method might still work. It would be helpful to show examples of how RRA fails under high MOI (even in supplement).

9. Reciprocally, it would be helpful to show why is RRA necessary if LR can overcome the problem of high MOI? What would the results from Fig 1b (target gene knockout) look like using LR instead of RRA tool?

*Response: Under high MOI condition, one cell may have multiple sgRNAs targeting multiple genes. Using RRA may lead to false positives, as cells that are in the front of the ranked list whose gene down- (or up-) regulation may come from other genes. An example is provided in Supplementary Fig. 11 (also in Reviewer-Only Fig 7 below), where almost all of the hits have very strong p values in the K562 high MOI dataset. Instead, the distribution of LR scores are centered on zero, an indication that LR module well separates the effect of genes at high MOI condition.*

*The advantage of scMAGeCK-RRA is it is a non-parametric approach and it is able to detect non-linear perturbations including multimodal effects (e.g., the only a fraction of cells have strong perturbation effect). An example is provided in Reviewer-Only Fig. 8 (also in Supplementary Fig. 3b), where CHEK1 perturbed cells only affect a small fraction of cells, as is seen in the red dots on the top. RRA can detect this change (the top hit in Fig. 2b), and CHEK1 is a known essential gene from DepMap data (in Reviewer-Only Fig. 3). However, LR is not able detect such subtle change (score=-0.06, adjusted p value=0.93). Another advantage of scMAGeCK-RRA is that it reaches lowest false positive rate compared with scMAGeCK-LR and MIMOSCA in the permutated data, as is demonstrated in the new Fig. 1e.*

*Based on these reasons, we used RRA to test target gene knockout for Figure 1b, except for K562 where LR is used. The results of Fig. 1b using LR in MCF10A and K562 are presented in Reviewer-Only Fig. 9. As expected, LR has a lower sensitivity than RRA especially in T cell datasets, if we compare it with Figure 1b. We thank the reviewer for the critical comments. We demonstrated the advantage and limitation of scMAGeCK-RRA/LR more clearly in the new Table 1, and revised the corresponding part in the discussion section as follows:*

*“The two modules of scMAGeCK provide complementary tools to study gene perturbation in different contexts. scMAGeCK-RRA is an algorithm that reaches the lowest false positive rate (Fig. 1e), and is able to detect subtle, non-linear expression changes that scMAGeCK-LR is not able to identify. For example, scMAGeCK-LR failed to detect CHEK1 (score=-0.06, adjusted p value=0.93) whose knockout reduces MKI67 expression in only a small fraction of cells (Supplementary Fig. 5b), which is readily identified as the top hit in scMAGeCK-RRA (Fig. 2b). In contrast, scMAGeCK-LR provides a convenient tool to model the expressions of all genes and deals with cells infected by multiple sgRNAs, where scMAGeCK-RRA may fail (e.g., in Supplementary Fig. 11).”*

10. In Fig 3b, why is RASA2 not shown as a negative regulator of proliferation under "stimulating" conditions as described in the text? This should be clarified.

*Response: RASA2 is a negative regulator in stimulating conditions, but the FDR value is not significant (0.37). Therefore, it’s not included in Fig. 3b. We clarified this at the end of the section “Investigating multiple phenotypes using scMAGeCK” as follows:*

*“Some genes may have opposite roles in different conditions; for example, RASA2 is a positive regulator of MKI67 in non-stimulating cells (Fig. 3b). On the other hand, genome-wide screens on stimulating T cells revealed RASA2 as a negative regulator (Fig. 2e). This is consistent with CROP-seq data, although the FDR is not significant (0.37 in stimulating T cells; Fig. 2c).”*

11. Could the authors comment on the performance of Lin28a and Lin28b in their CROPseq experiment? Both genes were not validated as knocking out their target by RRA analysis. Yet each shows a specific clustering pattern in Supp Fig 1. Does this mean that knocking out these genes leads to a strong effect on a rare subset of cells?

*Response: Knocking out Lin28a/b indeed led to a strong effect on a rare subset of cells in Supplementary Fig. 8 (the original Supplementary Fig. 1). We suspect the reason may be (1) their knockouts may not change gene expressions, and (2) there may be potential off-target issues for sgRNAs targeting both genes. We added explanations to the “scMAGeCK identified key genes associated with different pluripotency states of embryonic stem cells” section as follows:*

*“Interestingly, knocking out Lin28a/b did not change their expressions, but led to a strong effect on a subset of cells (Supplementary Fig. 8), possibly due to the potential off-target effects of these sgRNAs, or the possibility that their knockouts do not affect Lin28a/b expressions.”*

12. Again it would be helpful for authors to try to understand what determined the successes vs failures to detect target genes in Fig 4c. The failure rate seems quite high. Would more guides per target gene help? Showing the expression vs selection score for the ES cell data generated by authors could be informative.

*Response: The expression vs. selection score is presented in the original Figure 5a. For the hits in Figure 4c, 8 out of 15 (53%) genes reached statistical significance (success). For the remaining 7 hits, two are slightly over the FDR 0.1 cutoff (Klf4: 0.13; Mthfd1: 0.17). Six out of the seven hits have less than 100 supporting single cells, which may contribute to the failures to detect target gene expression reduction. As is also mentioned in our response to comments #3, #4 and #11, their knockouts may not necessarily change their expressions, and the sgRNA efficiencies may be low. All these factors contribute to the failures reported in Figure 4c. We discussed possible reasons of failures in Fig 4c in the section “scMAGeCK identified key genes associated with different pluripotency states of embryonic stem cells” as follows:*

*“For the remaining seven genes that do not reach FDR threshold (0.1), six have less than 100 supporting single cells. The small number of single cells, together with other reasons (e.g., low sgRNA efficiency, gene knockout does not change their expression), may contribute to the “failure” to detect target gene down regulation.”*

We didn’t investigate the effect of the number of guides per target gene, since this number is still small for all the CROP-seq datasets we currently have (<4 guides/genes).

13. Fig 4e. What are the inputs and outputs for the network analysis? Would it be feasible to input all 15 target genes and discover all significant relationships with marker genes? This would seem the most "agnostic" way to look at gene networks.

*Response: The inputs are the results of scMAGeCK that tests all 15 gene perturbations on a set of expression markers that are known to be specific to each state (i.e., Nanog and Klf4 for naïve state, and Otx2 for primed state in Fig. 4e). The outputs are the significant target-marker regulations identified in scMAGeCK, which are used to draw the network. We added the explanations of network analysis at the last paragraph of the section “scMAGeCK identified key genes associated with different pluripotency states of embryonic stem cells” as follows:*

*“The inputs of the network analysis are scMAGeCK-RRA results, using a set of predetermined expression markers that are specific each state (i.e., Nanog and Klf4 for naïve state, and Otx2 for primed state in Fig. 4e). Target-marker associations with statistical significance (FDR <0.01) are used to draw the network.”*

14. How do the results of the network analysis change as the number of marker genes increases? E.g. sometimes examples are shown with one marker per phenotype, but other times with multiple markers per phenotype. It is unclear why different numbers of markers per phenotype are used, and how a user should select marker genes. Also how many phenotypes to select?

*Response: As the number of marker genes increases, fewer edges may be identified since more p values needed to be adjusted. The number of phenotypes and markers will be mainly selected by users, based on biological knowledge.*

15. It is helpful that the authors show the benefit of high MOI. It would also be helpful if the authors could provide some discussion on hypotheses as to why high MOI helps? Is there any tradeoff to high MOI? How to pick best MOI for prospective experiments?

*Response: We emphasized the reason why high MOI helps in the newly added paragraph at the end of the section titled “High target expression and high MOI improves the power of single-cell CRISPR screening”, mainly due to the higher number of single cells per target gene that increases the statistical power:*

*“Third, high MOI increases the number of single cells per target gene, one reason that the statistical power is improved compared with low MOI.”*

*One potential limitation is that high MOI may not be suitable for CRISPR-knockout based CROP-seq, the most common type of CROP-seq. This is because multiple DNA cleavage may likely to induce strong DNA damage response in the cells, a factor that has been discussed extensively in CRISPR knockout screens (Wang et al., 2015; Meyers et al., 2017; Morgens et al., 2017). Since there’s only one CROP-seq dataset that uses high MOI condition, we are not able to draw any conclusion on the best MOI. We added the following paragraph in the discussion section:*

*“Compared with CROP-seq using low MOI condition, high MOI reaches a better performance in terms of target gene knock down (Fig. 5d-f). However, high MOI condition may not be suitable for CRISPR knockout based CROP-seq, as multiple DNA cleavage within single cell may induce strong DNA damage response in the cells (Wang et al., 2015; Meyers et al., 2017; Morgens et al., 2017). Further investigations are needed to determine the best MOI for CROPseq based on CRISPR/Cas9 gene knockouts.”*

16. It is helpful that the authors show the challenge of lowly expressed genes. Any strategies to deal with low expressed genes?

*Response: We added possible approaches to deal with lowly expressed genes in the discussion section, including (1) using CRISPR inhibition (CRISPRi) instead of CRISPR knockout; (2) increasing the number of single cells per target gene, and (3) looking at known downstream genes instead of the target gene itself:*

*“Most of the CROP-seq datasets based on target gene knockout have a low successful rate (Fig. 1b). There may be various reasons to the failures, including (1) low target gene expression, (2) low guide knockout efficiency, and (3) not enough single cells to reach a statistical significance. Therefore, to increase the success rate, users may pick up genes with moderate or high expression, and/or ensure that there are enough number of cells for the analysis (e.g., by increasing MOI). One caveat of this approach is that target gene knockout may not necessarily reduce its expression (Hill et al. 2018). To overcome this limitation, one may look at the expression of known downstream targets, or switch to CRISPR inhibition instead of CRISPR knockout to directly repress target gene expression. As more CROP-seq (or other types of single-cell technology) datasets accumulate, we may be able to study how guide knockout efficiency affect the outcome of the screen.”*

17. In the Results section "scMAGeCK method overview" 3rd paragraph, 3rd line, should be "LR" not "RRA".

*Response: We corrected the typo in the method overview section.*

Reviewer #3: In this manuscript the authors develop a new single-cell CRISPR screening analysis pipeline aka scMAGeCK. They validate this pipeline across multiple experiments: one, identifying genes and enhancers that modulate expression of MKI67 (Ki-67); two, a single-cell CRISPR screen on mouse embryonic stem cells (mESC) to identify genes associated with pluripotency states; and three, exploring key factors that improve the statistical power of single-cell CRISPR screens. Overall the study is comprehensive and well executed. However, as multiple computational pipelines exist for analyzing perturb-seq like screens, the authors must benchmark their tools efficacy vis-a-vis these especially the Regev et al and Weissman et al analysis workflows (similar to table 1 of: https://genomebiology.biomedcentral.com/articles/10.1186/s13059-014-0554-4). A side-byside *comparison would enable broader adoption of the scMAGeCK pipeline.*

*Response: We thank the reviewer for the positive comments and critical suggestions on the pipeline comparison. As is also raised by Reviewer #1, we compared scMAGeCK with two existing softwares: MUSIC and MIMOSCA, and presented the results in the new section of “Comparisons with clustering analysis and other algorithms” (also in our response to comment #1 of Reviewer #1). We did not compare Weissman et al., as there is no standalone software available for us to run CROP-seq datasets.*

*A comparison of the features of these pipelines is presented in the Table 1 (similar to table 1 of the MAGeCK paper), and in Reviewer-Only Table 1 below.*

**Second round of review**

**Second round of review**

**Reviewer 1**

In their revised manuscript “Linking genotypes with multiple phenotypes in single-cell CRISPR screens”, Yang et. al. have performed a number of new analyses to thoroughly address the concerns that were raised in the initial round of review. The work now shows that scMAGeCK is a computational tool to identify genotype-phenotype relationship from scCRISPR screens. We have a few minor questions/suggestions in considering the manuscript further.

1)     For Fig 2F examining the enrichment of essential genes using Ki-67, majority of genes annotated as essential genes do not statistically show dependency using the Ki-67 markers. How do non-essential genes perform in these analyses? Is there clear separation between the essential and non-essential genes?

2)     We thank the authors for cross-analyzing their analyses to the AVANA CRISPR screens. We agree with the authors that some tumor suppressors and oncogenes may behave differently depending on the cancer types as Supplementary Figure 5C is not informative for majority of the genes except CHEK1 and RAD51. As MCF10A was not screened in by CRISPR in the AVANA dataset, the authors were not able to perform the comparison for that particular cell line; however, RNAi screens in MCF10A are available (although they do not include all these genes) where the dependencies for these strong tumor suppressors and oncogenes should behave similarly between these two perturbation technologies and would allow for a resolution of certain discrepancies.

**Reviewer 2**

The authors have adequately responded to my prior concerns. This method for analysis of scRNA-seq based CRISPR screens will be useful for the functional genomics community.

**Authors Response**

**Point-by-point responses to the reviewers’ comments:**

Reviewer #1: In their revised manuscript “Linking genotypes with multiple phenotypes in single-cell CRISPR screens”, Yang et. al. have performed a number of new analyses to thoroughly address the concerns that were raised in the initial round of review. The work now shows that scMAGeCK is a computational tool to identify genotype-phenotype relationship from scCRISPR screens. We have a few minor questions/suggestions in considering the manuscript further.

1)	For Fig 2F examining the enrichment of essential genes using Ki-67, majority of genes annotated as essential genes do not statistically show dependency using the Ki-67 markers. How do non-essential genes perform in these analyses? Is there clear separation between the essential and non-essential genes?

*Response: We look at the distribution of MKI67 selection scores between essential and non-essential genes and presented the results in the new Figure 2f inset also in the Reviewer-Only Figure 1 below. The selection score of non-essential genes are much smaller, and there’s a clear separation between two groups (p=2.85e-9 using Wilcox rank sum test).*

**Reviewer-Only Figure 1 (also in the new Figure 2f inset):** The distribution of MKI67 selection score between essential genes and other genes. P value is calculated using Wilcox rank sum test.

**Reviewer-Only Figure 1 (also in the new Figure 2f inset):** The distribution of MKI67 selection score between essential genes and other genes. P value is calculated using Wilcox rank sum test.

**Reviewer-Only Figure 1 (also in the new Figure 2f inset):** The distribution of MKI67 selection score between essential genes and other genes. P value is calculated using Wilcox rank sum test.

2)	We thank the authors for cross-analyzing their analyses to the AVANA CRISPR screens. We agree with the authors that some tumor suppressors and oncogenes may behave differently depending on the cancer types as Supplementary Figure 5C is not informative for majority of the genes except CHEK1 and RAD51. As MCF10A was not screened in by CRISPR in the AVANA dataset, the authors were not able to perform the comparison for that particular cell line; however, RNAi screens in MCF10A are available (although they do not include all these genes) where the dependencies for these strong tumor suppressors and oncogenes should behave similarly between these two perturbation technologies and would allow for a resolution of certain discrepancies.

*Response: We thank the reviewer to point this out. We found seven out of eight genes (except CDH1) are included in MCF10A RNAi screens in DepMap (Reviewer-Only Figure 2, also Additional file 1: Figure S5d). We found six out of seven gene scores are consistent with Figure 2a-b: two tumor suppressors (TP53, ARID1B) have positive dependency scores, and four out of four oncogenes (CHEK1, GATA3, CASP8 and RAD51) have negative scores. The only inconsistent one is RUNX1.*

**Reviewer-Only Figure 2 (also in the new Additional file1: Figure S5d):** The dependency scores of top hits in Fig. 2a-b in MCF10A RNAi screens in DepMap. CDH1 was not screened in RNAi screening dataset.
